# Introgression of exotic *Cervus* (*nippon* and *canadensis*) into red deer (*Cervus elaphus*) populations in Scotland and the English Lake District

**DOI:** 10.1002/ece3.3767

**Published:** 2018-01-22

**Authors:** Stephanie L. Smith, Helen V. Senn, Sílvia Pérez‐Espona, Megan T. Wyman, Elizabeth Heap, Josephine M. Pemberton

**Affiliations:** ^1^ Institute of Evolutionary Biology School of Biological Sciences University of Edinburgh Edinburgh UK; ^2^ WildGenes Laboratory Royal Zoological Society of Scotland Edinburgh UK; ^3^ Estación Biológica de Doñana CSIC Seville Spain; ^4^ Mammal Vocal Communication and Cognition Research School of Psychology University of Sussex Falmer UK

**Keywords:** conservation, introduction, microsatellite, hybridization, mtDNA, red deer, sika, wildlife management

## Abstract

Since the mid‐19th century, multiple introductions of Japanese sika deer (*Cervus nippon nippon*) and North American wapiti (*C. canadensis*) have taken place in the British Isles. While wapiti have generally been unsuccessful, sika have been very successful, especially in Scotland where they now overlap at least 40% of the range of native red deer (*C. elaphus*). Hybridization between these two species and red deer has been demonstrated in captivity and in the wild. Using a panel of 22 microsatellite loci that are highly diagnostic between red deer and sika, and moderately diagnostic between red deer and wapiti, we investigated the extent of introgression between these species in 2,943 deer sampled from around Scotland and from the English Lake District using the Bayesian clustering software STRUCTURE. We also used a diagnostic mitochondrial marker for red deer and sika. Our survey extends previous studies indicating little introgression of wapiti nuclear alleles into red deer, in particular in Northern Scotland, Kintyre, and the Lake District. We found a new area of extensive sika introgression in South Kintyre. In the North Highlands, we show for the first time geographically scattered evidence of past hybridization followed by extensive backcrossing, including one red‐like individual with sika introgression, two sika‐like individuals with red deer introgression, and six individuals that were apparently pure sika at the nuclear markers assessed but which carried red deer mitochondria. However, there has not been a collapse of assortative mating in this region. Similarly, in the English Lake District red deer, we found only traces of past sika introgression. No sika alleles were detected in the Central Highlands or the Hebridean red deer refugia. We make suggestions for management to prevent further spread of sika alleles into red deer and *vice versa*.

## 1 INTRODUCTION

Hybridization is the interbreeding of genetically distinct taxa, and introgression is the gene flow between taxa which can occur thereafter (Mallet, 2005). While hybridization can and does occur naturally, anthropogenic activity can bring species into sympatry that historically have not been in contact over appreciable evolutionary time and lead to anthropogenic hybridization and introgression (Allendorf, Leary, Spruell, & Wenburg, 2001). Examples of such activity include trade and transport of domestic animals and crops, introduction of exotic species, habitat modification and degradation, and land management for recreational activities (Leary, Allendorf, & Forbes, 1993; Rhymer, Williams, & Braun, 1994). When introgression occurs to the extent that phenotypes become intermediate between two species, assortative mating may be compromised and the system can collapse into a “hybrid swarm,” as has occurred, for example, between introduced mallards and Native gray ducks (*Anas spp*.) in New Zealand (Rhymer et al., 1994), and between cutthroat trout (*Oncorhynchus spp*.) in North America (Trotter & Behnke, 2008).

**Figure 1:**
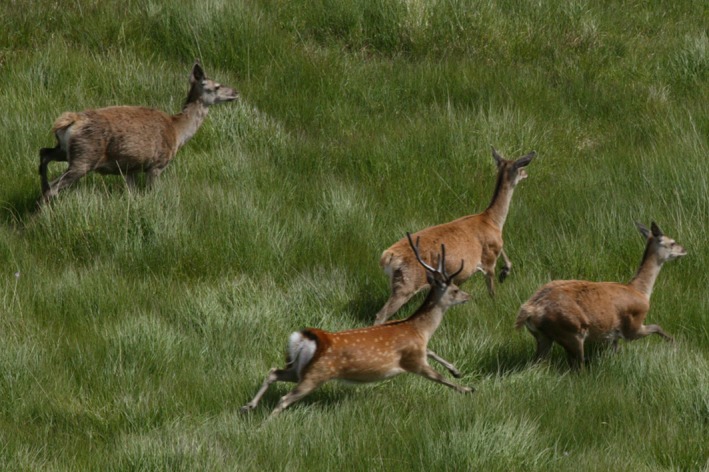
A sika male photographed running with red deer females during a helicopter deer count in the North of Scotland in 2006. Photograph Deer Commission for Scotland (now Scottish Natural Heritage)

In this article, we address anthropogenic hybridization and introgression among genus *Cervus* deer in the British Isles, focusing on the red deer, which is native to Europe, the Japanese sika and the North American wapiti (respectively, *Cervus elaphus*,* C. nippon nippon* and *C. canadensis*). Based on mitochondrial sequence information, the progenitor of these species originated from around Kyrgyzstan (Ludt, Schroeder, Rottmann, & Kuehn, 2004; Pitra & Lutz, 2005). A western‐migrating clade became the medium‐sized red deer while those moving east bifurcated into the larger wapiti and the smaller sika, which itself diversified into several subspecies throughout south‐eastern Asia including the Japanese sika (*C. n. nippon*) (Cook, Wang, & Sensabaugh, 1999; Kuwayama & Ozawa, 2000; Ludt et al., 2004). A recent analysis puts the split between the red deer lineage and the sika/wapiti lineage at 6MY BP (Lorenzini & Garofalo, 2015). While red deer and sika differ in body size (red deer are larger ; see Figure 1), a variety of other phenotypic traits (see [Senn & Pemberton, 2009] Table 1), and chromosome number (red 2N = 68; sika 2N = 64 [Herzog & Harrington, 1991]), they are known to have hybridized both in captivity and in the wild (Goodman, Barton, Swanson, Abernethy, & Pemberton, 1999; Harrington, 1973; Huang, Chi, Nie, Wang, & Yang, 2006; Lowe & Gardiner, 1975; Senn & Pemberton, 2009). Similarly, red deer and North American wapiti differ in body size (wapiti are larger) but are known to hybridize in captivity and the wild (Moore & Littlejohn, 1989; Shackell, Drew, Pearse, & Amer, 2003).

**Table 1:** Sample sizes, deer stalker‐assigned phenotypes, and genetic dataset completeness for the 2,943 individuals successfully genotyped (for at least 20 of the 22 markers), shown for the five regions sampled and the wapiti controls

Study area	*n* Individuals	*n* Phenotypic red	*n* Phenotypic sika	*n* Phenotypic wapiti	*n* Phenotypic hybrid animals	*n* Not assigned phenotype	Microsatellite dataset (% complete)	MtDNA dataset (% complete)
Kintyre, Scotland	1,054	677	314	0	32	31	99.71	98.96
Central Highlands, Scotland	406	368	1	0	0	37	98.9	99.75
Hebrides, Scotland	727	727	0	0	0	0	97.49	100
North Highlands, Scotland	570	256	206	0	35	73	99.78	99.65
Lake District, England	137	137	0	0	0	0	98.77	100
Canada	49	0	0	49	0	0	99.63	NA

The native red deer of the British Isles fall into the western (rather than Eastern) European mitochondrial clade of red deer (Krojerova‐Prokesova, Barancekova, & Koubek, 2015; Pérez‐Espona, Pérez‐Barbería, et al., 2009) and have in the past been placed in their own subspecies, *Cervus elaphus scoticus,* although this and other subspecies designations are not generally supported these days. The fortunes of the population have fluctuated over historical times, and nowadays the largest concentration of red deer is in Scotland with smaller, more isolated populations in England, of which the Lake District population is one. Within Scotland, there are an estimated 350,000 red deer living on open hill land and an unknown number in woodland (Clutton‐Brock, Coulson, & Milner, 2004). Unfortunately, since the mid‐19th century, there has been a series of deliberate and accidental introductions of exotic deer, including other red deer subspecies from Europe, the North American wapiti and Japanese sika, creating many opportunities for hybridization with red deer. The genetic influence of mainland European red deer translocations on Scottish red deer appears to have been generally low (Pérez‐Espona, Pérez‐Barbería, et al., 2009; Pérez‐Espona et al., 2013). In this article, we focus on the potential for wapiti and sika introgression.

North American wapiti were introduced to a few sites in Scotland, mostly in enclosures, in attempts to increase body and antler size, and hybridization is reported to have occurred (Whitehead, 1964). The first individual introduced to Scotland is thought to have been to Dunkeld, Perthshire in the early 1800s, by a former Duke of Atholl (Whitehead, 1964). During the 1890s, wapiti were introduced and hybridized with red deer at Monymusk, Aberdeenshire, and in the early 1900s, this herd (c. 30 animals) was translocated to Mamore forest, Inverness‐shire (Whitehead, 1964). In England, wapiti were introduced to Derby around the 1790s, and herds were kept in Woburn Park (Bedfordshire), Buckinghamshire, Kent, Sussex and Northamptonshire and, around the turn of the 20th century, at Rigmaden Park, near the English Lake District (Whitehead, 1964). Pure wapiti do not appear well adapted to the British environment, and there are currently few in captivity and none in the wild in the British Isles. They are highly susceptible to lungworm and, in comparison with red deer, wapiti females have delayed maturity and wapiti males exhibit lower levels of aggression during the rut (Asher et al., 2005; Pérez‐Espona, Pérez‐Barbería, & Pemberton, 2011).

**Figure 2:**
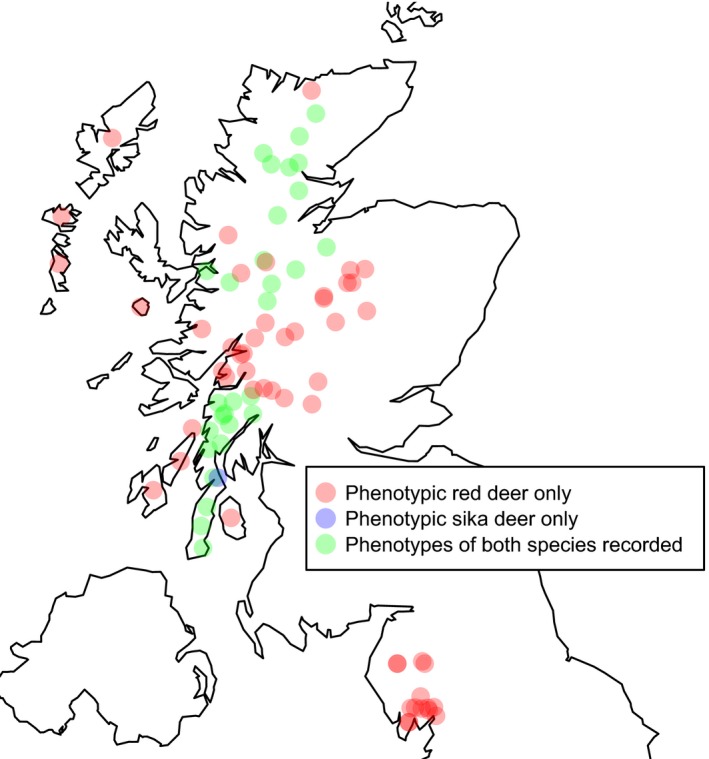
Map of the study areas in Scotland and England; red indicates areas from where only phenotypically red deer were sampled, green indicates areas where only phenotypically sika were sampled and blue are where both species were sampled. Wapiti samples to assess past introgression between wapiti and red deer were obtained from Canada

Numerous introductions of sika as an ornamental species in deer parks have also taken place throughout the British Isles. Historical records (Ratcliffe, 1987), skull morphology (Lowe & Gardiner, 1975), and genetics (Goodman et al., 2001) suggest that the vast majority of the resulting wild sika in the British Isles are Japanese sika. Specifically, British Isles sika have mtDNA sequences similar to those of sika on Kyushu, which is consistent with the fact that the port of Nagasaki on Kyushu was the only port open for export from Japan at the time of the importations (Goodman et al., 2001). In contrast to wapiti, sika are very successful in the British Isles, especially in the habitat provided by commercial forestry. The distribution of sika in Scotland is attributed to 12 separate episodes of deliberate release or escape (Pérez‐Espona, Pemberton & Putman 2009; Ratcliffe, 1987), and it is estimated that they occupy around 14,000 km^2^ of Scotland. Due to the difficulty of counting sika, there are no population estimates, but about 7,000 are currently culled annually in Scotland with little apparent impact on the population. Sika have a much patchier distribution in England (Diaz, Hughes, Putman, Mogg, & Bond, 2006; Pérez‐Espona, Pemberton et al., 2009; Ratcliffe, 1987; Ward, 2005). Inevitably, the spread of sika has led to extensive range overlap with that of the native red deer (at least 40% of the red deer range in Scotland (Livingstone, 2001); see Figure 2) and has provided an opportunity for hybridization and introgression, which has been documented at the phenotypic level in the wild (Ratcliffe, 1987). Sika have also been introduced to many places in England. Following the introduction of Japanese sika during the late 1800s to Rigmaden Park and another site near the Lake District, phenotypic hybrids with red deer were reported and confirmed using craniometrics (Lowe & Gardiner, 1975; Ratcliffe, 1987). The nearby red deer population in Grizedale is thought to be one of the few remaining of native English descent, and its genetic integrity would be at risk from nearby hybrids (Pérez‐Espona, Pemberton et al., 2009). Japanese sika have also been introduced to other parts of Europe where phenotypic hybrids have been documented in Ireland (Harrington, 1973) and the Czech Republic (Bartos, Hyanek, & Zirovnicky, 1981; Bartos & Zirovnicky, 1981). We note here that Asiatic mainland subspecies of sika have also been introduced to England and Ireland (Whitehead, 1964). However, there is no concrete evidence of their introduction to Scotland or Cumbria (Powerscourt, 1884; Ratcliffe, 1987; Whitehead, 1964), and therefore, these subspecies are not addressed further in this article.

Genetic analysis can greatly improve the understanding of hybridization and introgression, and one of the best‐studied examples of red‐sika hybridization in the wild using genetics is in Kintyre, Argyll, Scotland. Two male and nine female sika were introduced at Carradale, near to the southern end of this peninsula in 1893 (Ratcliffe, 1987). The herd expanded into sympatry with red deer, and hybrids were subsequently observed (Ratcliffe, 1987; Whitehead, 1964) and confirmed genetically (Goodman et al., 1999). More recently, Senn and Pemberton (2009) analyzed 735 red deer and sika sampled from throughout Kintyre and, using 22 highly diagnostic microsatellite markers and a diagnostic mitochondrial marker, found that while hybrids were generally rare, at one site, West Loch Awe (WLA), 43% of sampled deer were hybrids. At WLA, the normal assortative mating patterns appear to have broken down, producing a hybrid swarm. The pregnancy rate of female hybrids at WLA does not differ from that of either parental species in the same area, suggesting hybrids are as fertile as the parental species (Senn, Swanson, Goodman, Barton, & Pemberton, 2010).

Regarding other parts of Scotland, genetic work on red deer sampled from the Central Highlands found no evidence for the presence of introgressed mtDNA haplotypes from wapiti or Japanese sika (Pérez‐Espona, Pérez‐Barbería, et al., 2009), no evidence of wapiti Y chromosomes (Pérez‐Espona et al., 2011) and little evidence of wapiti nuclear marker introgression in a study that also included Hebridean red deer populations (Pérez‐Espona et al., 2013). In parts of the North Highlands, a study using 10 of the microsatellite markers used here, genotyped using autoradiography and analyzed with less powerful procedures suggested extensive, low‐level, introgression of red deer alleles into sika (Swanson, 2000). However, large parts of Scotland remain unscreened for red‐sika hybridization using an appropriately powerful panel of diagnostic markers.

The only genetic study of red deer‐sika hybridization in England to date used a small panel of microsatellite markers on samples from the New Forest, Hampshire, and Dorset and concluded that despite evidence of low‐level introgression of red nuclear DNA into sika in some areas, there was no indication of recent hybridization and mating between the species is strongly assortative at these sites (Diaz et al., 2006).

Given red‐sika hybridization occurs, many traits could introgress between the species, altering their appearance, ecology, and life history, with implications for deer management. Lowe and Gardiner (1975) reported intermediate craniological morphology for putative hybrids in the Lake District, and in a well‐documented hybrid swarm in County Wicklow (Ireland), Harrington (1973) observed a general merging of size and pelage of animals in the region. Similarly, vocalizations of red deer‐sika hybrids are intermediate between parental species (Long, Moore, & Hayden, 1998; Wyman, Locatelli, Charlton, & Reby, 2014). Carcass weight, jaw length, and incisor arcade breadth are all greater in sika‐like hybrids than in “pure” sika and lower in red deer‐like hybrid females than in “pure” red deer females (for definition of “pure”; see Senn, Swanson et al. (2010)). Overall, phenotypic modifications such as these highlight the substantial additive genetic variation for quantitative traits in hybrid deer and the potential for introgression of traits associated with dispersal, fertility, and elusiveness to exacerbate effective identification and management of introgressed deer populations and the risk of hybrid swarms occurring.

In this study, we sampled deer over a much greater area of Scotland than hitherto, and in the English Lake District, and analyzed the samples with a more powerful genetic approach for red‐sika hybridization than previous surveys (except [Senn & Pemberton, 2009]) in order to document the impact of exotic *Cervus* deer on the red deer populations of Scotland and the English Lake District. The specific objectives were as follows:

1. To assess the current extent of hybridization and introgression between introduced *Cervus* deer and red deer across Scotland and in the Lake District.
2. To determine, if possible, the initial direction of hybridization.
3. To suggest future management actions that could prevent further hybridization and introgression.

## 2 MATERIALS AND METHODS

### 2.1 Study area and sampling

The study area consisted of five regions, four in Scotland and one in England (Figure 2), including many areas of sympatry between red deer and sika (Ward, 2005). From **Kintyre**, Argyll, we included genotype data at the study loci from the previous study of Senn and Pemberton (2009) (*n* = 735 individuals) and we collected additional samples, specifically from WLA and South Kintyre (SK) in 2008‐11, bringing the total number of animals sampled from Kintyre to 1,054. Many of the islands in the **Hebrides** were designated as refugia for native red deer in 1999 to protect them from introgression from other *Cervus* species (Wildlife and Countryside Act [variation of schedule 9] Order 1999). This designation requires regular assessment of the Hebridean populations for sika introgression in order to confirm their status. We included samples from 727 animals from eight Hebridean islands collected in 2009–2010. For this study, 570 individuals from 18 Forestry Commission Scotland management units across the **North Highlands** were sampled in 2009–2011. Samples obtained from the ***Central Highlands*** included a set from in and around the Cairngorms National Park and the Loch Lomond and the Trossachs National Park (*n* = 171 individuals, collected 2008–2012) and a set from open hill estates across the Central Highlands from the study of Pérez‐Espona et al. (2008) (*n* = 235, collected 2003—2004) giving a total of 406 animals sampled. Finally, 137 samples were obtained from the **English Lake District** via Eleni Socratous, University of Leicester. These samples were collected during 2008–2010 and mostly came from Grizedale. Samples from 49 North American wapiti were provided by Prof. D.W. Coltman, University of Alberta, Canada.

All new samples were obtained during the course of normal deer culling operations, and deer species were shot in the proportions in which they were encountered. Stalkers identified all sampled deer from the Hebrides and the Lake District as red deer. Deer sampled from the Central Highlands were mainly identified as red deer but with a few sika in the more recently collected set. Samples from Kintyre and the North Highlands were identified as red deer, sika, or hybrid. The Scottish samples consisted of a mix of frozen ear tips, frozen jaws (including adherent muscle), and ear tips in 99% ethanol. The Lake District samples consisted of frozen tissue. The wapiti samples were provided as extracted DNA.

### 2.2 DNA analysis

Genomic DNA was extracted from tissue samples with the DNeasy Blood and Tissue Kit (Qiagen) or DNAse Spin Tissue Mini kit (Bioline), both according to the manufacturer's instructions. Individual samples were genotyped at a panel of 22 diagnostic microsatellite markers following previously published protocols (Goodman et al., 1999; Senn & Pemberton, 2009), the details of which are given in Appendix Table S1. Originally derived from cattle (*Bos taurus*) and sheep (*Ovis aries*), these markers were selected because when used to genotype 44 red deer and 44 sika from diverse geographical locations, these species shared no common alleles (Goodman et al., 1999; Slate et al., 1998). In addition, they have some discriminatory power between red and wapiti (10/22 with large allele frequency differences). PCR products were run on an ABI3730 capillary sequencer (Applied Biosystems), using the internal standard Genescan LIZ 500 (Applied Biosystems). Fragment analysis was carried out using GeneMapper version 4.0 (Applied Biosystems).

Samples were also screened for their haplotype at the mitochondrial control region, which in *Cervus* spp. includes a 39‐bp sequence with a variable number of tandem repeats: Red deer have a single repeat while Japanese sika have three repeats (Cook et al., 1999). Amplification followed a published protocol (Cook et al., 1999) and repeat number was determined by assay on 4% agarose gels stained with ethidium bromide (Goodman et al., 1999) following which red deer have a 350‐bp band and Japanese sika a 430‐bp band.

### 2.3 Data analysis

The Bayesian clustering software structure 2.3.3 (Falush, Stephens, & Pritchard, 2003, 2007; Pritchard, Stephens, & Donnelly, 2000) was used to analyze the extent of individual and population admixture using the microsatellite genotype data in a number of separate **datasets**. In **analysis 1**, all red deer and sika samples from Scotland and Cumbria together with the 49 wapiti were analyzed to resolve the most likely population structure involving all three species (*n* = 2,943). In **analysis 2**, the wapiti samples and any individuals showing evidence of wapiti introgression were excluded in order to assess the extent of red‐sika hybridization separately (*n* = 2,887). In** analysis 3**, all “pure” sika and red‐sika hybrids were removed such that only red deer, wapiti, and their potential hybrids remained, to identify the presence of any introgression between these two species separately (*n* = 2,230). The number of inferred, genetically distinct populations (*K*) that maximizes the likelihood (Ln Pr(*X*|*K*)) of the dataset, assuming Hardy–Weinberg equilibrium and linkage equilibrium, was estimated by running five independent replicates at different values of *K* (1–8). The smallest value of *K* with the highest log likelihood (Ln Pr(*X*|*K*)), prior to it plateauing, was selected as the most likely (Pritchard et al., 2000). An alternative for estimating the best value of *K*, estimating the maximum rate of change in the log probability of the data between consecutive values of *K* (∆*K*), was also used to indicate the appropriate value of *K* (Evanno, Regnaut, & Goudet, 2005). All analyses were run with the same parameters as previous studies (Senn & Pemberton, 2009), namely the standard model of admixed ancestry (with the parameter α inferred from the data, using a uniform prior) and the model of correlated allele frequency (λ = 1), a burnin of 5 × 10^4^ and a run length of 10^6^ Markov chain Monte Carlo steps. This is within the suggested parameter values of Pritchard et al. (2000) and runs appeared to converge correctly. Null alleles can cause deviation from Hardy‐Weinberg equilibrium by causing a systematic pattern of missing genotype data and can cause errors in the estimation of *Q* (Falush et al., 2003; Senn & Pemberton, 2009). The frequency of null alleles was therefore estimated concurrently by incorporating a row of “999” values into the second line of the dataset and activating the option RECESSIVE ALLELES = 1. This function enables structure to “suspect” particular alleles as null alleles if, for example, they exhibit allele‐specific PCR failure. structure then treats these suspected null alleles as recessive instead of missing data and estimates their frequency at each locus (Falush et al., 2007; Senn & Pemberton, 2009). structure output data were manipulated using the software distruct (Rosenberg et al., 2002) for illustrative purposes.

Analysis conducted in structure generated a *Q* value for each individual, which represents the estimated proportion of ancestry to each of *K* groups. When analyses are run at *K* = 2 (as is typical for hybridization between two taxa), the *Q* values for membership to one of the two ancestral populations can be used as an index of the hybrid status of an individual; here *Q* = 0 represents a sika (or wapiti if that is the comparison) and *Q* = 1, a red deer. Delimiting the proportion of admixture that qualifies as a hybrid is difficult, principally due to the possibility that at some loci there may be ancestral sharing of rare alleles in the taxa under consideration. Here, a hybrid was defined on the basis of nuclear markers as an individual returning a *Q* value of 0.05 ≤ *Q* ≤ 0.95 between two taxa, following previous practice (Senn & Pemberton, 2009) with the 90% probability intervals of *Q* consulted for specific individuals (see Section 2.1). Such animals were identified as a “nuclear hybrid” based on the microsatellite markers. Individuals outside these boundaries were defined as “pure,” although may still contain introgressed alleles beyond the detection limit of the markers, as 22 markers enable detection of only 2–3 generations of backcrossing with confidence for a specific individual (Boecklen & Howard, 1997). As it is highly unlikely that mtDNA control region types are shared through common ancestry, a hybrid was also defined as a “mitochondrial hybrid” if the mtDNA haplotype was discordant with a pure nuclear genotype (i.e., red deer mtDNA in animals with *Q* < 0.05 or sika mtDNA in animals with *Q* > 0.95). This latter type of hybrid indicates introgression beyond the resolution of the nuclear markers.

The direction of initial hybridization events (i.e., which taxon was the female parent) can only be assessed from cytonuclear data in F1 hybrids. An F1 individual should have a *Q* close to 0.5 in a *K* = 2 structure analyses and it should be heterozygous for taxon‐specific alleles at all loci. In order to determine whether we had sampled any F1 red‐sika hybrids, we examined the posterior allele frequencies for the parental taxa generated by structure following analysis 2 and assigned alleles as red deer specific, sika specific, or inconclusive, according to conservative criteria (Appendix Table S2). The genotypes of hybrids were re‐coded according to the origin of each allele at each locus to determine the proportion of loci that were red‐sika heterozygous relative to all loci genotyped in that individual, and this heterozygosity index was compared with *Q* for all individuals.

## 3 RESULTS

### 3.1 Genetic diversity

All individuals were successfully genotyped for at least 20 of 22 of the microsatellite loci (Table 1). Almost 9% of all individuals failed to amplify at the single locus TGLA337 (predominantly animals from the Hebrides), accounting for almost 40% of the missing data. Genetic diversity indices within each phenotypic class (red deer, sika, wapiti) are given in Table S3. Mean allelic diversity was highest in red deer (10.1), followed by sika (7.1), then wapiti (3.4).

### 3.2 Hybridization

#### 3.2.1 Analysis 1: Population structure assessment of all red deer, sika, and wapiti (*n* = 2,943)

The log likelihoods calculated in structure revealed that *K* = 2 was the smallest number of genetic clusters that best described the population structure, with an average Ln Pr (*X*|*K*) of −14603.8 (*SD* 10.7) and a rate of change of 2506.9 (Figure S1). At this value of *K*, as might be predicted from the choice of markers, red deer, and sika were differentiated, but not wapiti, which clustered with red deer (see Figure S2). At *K* = 3, the red deer subdivided, revealing that the red deer on the island of Harris and Lewis in the Hebrides were highly differentiated from all other red deer including wapiti. It was only at *K* = 4 (−140,655.48, *SD* 661.36; rate of change 7.7) that wapiti were differentiated from red deer and sika (Figure S3). To illustrate the three‐way admixture results, we merged the two red deer population clusters (Table 2, Figure S3 and Table S4). No three‐way hybrids between red deer, sika, and wapiti were detected (Table 2, categories 6, 9 and 12). Seven individuals showed wapiti introgression; these were red deer‐like individuals from Kintyre (*n* = 2), the Hebrides (*n* = 1), North Highlands (*n* = 2), and the Central Highlands (*n* = 2) (Figure S4c). Six of these individuals had 90% probability intervals that spanned zero; the exception was from the Central Highlands. This very low wapiti introgression was in contrast to the identification of 98 individuals classified as “red deer‐like hybrid with recent sika ancestry” (Table 2, category 4) and 78 “sika‐like hybrids with recent red deer ancestry” (Table 2, category 7). Overall 5.98% of individuals provided evidence for red‐sika hybridization, compared to only 0.24% of individuals for red‐wapiti hybridization.

**Table 2:** Admixture classification of all individuals, based on the *Q* values from analysis 1 in structure with *K* = 4 (red deer clusters I and II combined), based on a three species version of the classification approach of Senn and Pemberton (2009). Probability intervals were not used in these designations

Category	Estimated membership to sika	Estimated membership to wapiti	Category	Scotland				England	Total
				No. (& %) of animals from Kintyre	No. (& %) of animals from Central Highlands	No. (& %) of animals from the Hebrides	No. (& %) of animals from North Highlands	No. (& %) of animals from Cumbria	No. (& %) of animals from all sites
1	0 ≤ *Q* < 0.05	0 ≤ *Q* < 0.05	“Pure” red	618 (58.63)	398 (98.03)	726 (99.86)	299 (52.46)	134 (97.81)	2,175 (73.90)
2	0.90 < *Q* ≤ 1	0 ≤ *Q* < 0.05	“Pure” sika	265 (25.14)	6 (1.48)	0 (0)	265 (46.49)	0 (0)	536 (18.21)
3	0 ≤ *Q* < 0.05	0.90 < *Q* ≤ 1	“Pure” wapiti	0 (0)	0 (0)	0 (0)	0 (0)	0 (0)	49 (1.66)
4	0.05 ≤ *Q* < 0.50	0 ≤ *Q* < 0.05	Red‐like hybrid with recent sika ancestry	94 (8.92)	0 (0)	0 (0)	1 (0.18)	3 (2.19)	98 (3.33)
5	0 ≤ *Q* < 0.05	0.05 ≤ *Q* ≤ 0.50	Red‐like hybrid with recent wapiti ancestry	2 (0.19)	2 (0.49)	1 (0.14)	2 (0.35)	0 (0)	7 (0.24)
6	0.05 ≤ *Q* ≤ 0.95	0.05 ≤ *Q* ≤ 0.95	Red‐like hybrid with recent sika and recent wapiti ancestry	0 (0)	0 (0)	0 (0)	0 (0)	0 (0)	0 (0)
7	0.50 ≤ *Q* < 0.95	0 ≤ *Q* < 0.05	Sika‐like hybrid with recent red ancestry	75 (7.12)	0 (0)	0 (0)	3 (0.53)	0 (0)	78 (2.65)
8	0.50 ≤ *Q* < 0.95	0.05 ≤ *Q* < 0.50	Sika‐like hybrid with recent wapiti ancestry	0 (0)	0 (0)	0 (0)	0 (0)	0 (0)	0 (0)
9	0.50 ≤ *Q* < 0.95	0.05 ≤ *Q* ≤ 0.95	Sika‐like hybrid with recent red and recent wapiti ancestry	0 (0)	0 (0)	0 (0)	0 (0)	0 (0)	0 (0)
10	0 ≤ *Q* < 0.05	0.50 ≤ *Q* < 0.95	Wapiti‐like hybrid with recent red ancestry	0 (0)	0 (0)	0 (0)	0 (0)	0 (0)	0 (0)
11	0.05 ≤ *Q* < 0.50	0.50 ≤ *Q* < 0.95	Wapiti‐like hybrid with recent sika ancestry	0 (0)	0 (0)	0 (0)	0 (0)	0 (0)	0 (0)
12	0.05 ≤ *Q* < 0.95	0.50 ≤ *Q* < 0.95	Wapiti‐like hybrid with recent red and recent sika ancestry	0 (0)	0 (0)	0 (0)	0 (0)	0 (0)	0 (0)

#### 3.2.2 Analysis 2: Population structure assessment of all red deer and sika (*n* = 2,887)

**Figure 3:**
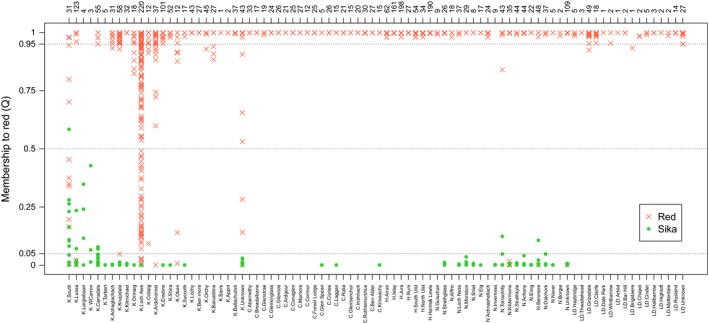
Membership values (*Q*) to red deer from analysis 2, as calculated by structure with *K* = 2 according to the site from which the individual was obtained (lower *x*‐axis) and the number of individuals sampled from each site (upper *x*‐axis). Scottish sites are plotted in an approximately south to north order, followed by the sample sites in the Lake District. Abbreviations represent main areas of sampling; K = Kintyre, C = Central Highlands, H = Hebrides, N = North Highlands and LD = Lake District. Red crosses and green dots indicate if the individual had a mtDNA haplotype characteristic of red deer or sika deer, respectively

As it is possible that the inclusion of wapiti genotypes could confound the analysis of red‐sika hybridization, in analysis 2, we repeated the analysis after removing the 49 wapiti samples and the seven red deer with evidence of wapiti introgression. The log likelihoods calculated in structure supported *K* = 2 as the smallest number of genetic clusters that describes the majority of the population structure, with an average Ln Pr(*X*|*K*) of 143907.42 (*SD* = 17.63) and rate of change of 3007.69 (Figure S5). Plots of *Q* values are given in Figures 3 and S6, with allele frequencies for population clusters at *K* = 2 given in Table S2. In practice, there was little detectable difference in the *Q* values obtained for each individual in this analysis and those obtained for the same individuals in analysis 1. Considering first **Kintyre**, in total, 617 (58.7%) of the deer from this area were pure red deer, 270 (25.6%) were pure sika, and 165 (15.7%) were hybrid. Specifically, within two regions of Kintyre, WLA and SK, 45% and 55%, respectively, of the individuals analyzed were nuclear hybrids. Sites adjacent to these two main areas of introgression also returned substantial numbers of hybrids (Figures 3 and S6).

All 726 individuals sampled from the **Hebrides** were pure red deer individuals (*Q* > 0.95); there was no evidence for recent hybridization with or introgression from sika in the islands forming the red deer refugia off Scotland's west coast (Figures 3 and S6).

The 568 individuals from throughout the **North Highlands** consisted of 299 pure red deer, 266 pure sika, and three hybrids. Two hybrids were sampled at Torrachilty, one red‐like with sika introgression, and one sika‐like with red introgression, and one hybrid was sampled at Benmore, which was sika‐like with red introgression (Figures 3 and S6). The *Q* values assigned to the two Torrachilty hybrids had 90% probability intervals that did not overlap one or zero, respectively, while the Benmore hybrid's probability intervals did overlap zero.

In the **Central Highlands**, samples obtained from in and around the national parks (*n* = 171) were free from hybridization as were the wider sample of deer from upland estates throughout the Central Highlands (*n* = 233) (*Q* ≥ 0.99, Figures 3 and S6).

Similarly, 134 of the 137 individuals from the **Lake District** (97.8%) were pure red deer and just three fell within our definition of a hybrid (one each from Grizedale, Brigsteere, and an unknown location in Cumbria; Figures 3 and S6). All three of these hybrids were red deer with low‐level sika introgression, and in two cases, the *Q* value had a 90% probability interval that did not overlap with one (one each from Grizedale and Brigsteere).

#### 3.2.3 Mitochondrial haplotypes in relation to analysis 2

Mitochondrial DNA analysis added further resolution to the structure analysis using nuclear markers (Figure 3; 2,868 Scottish deer had a resolved mtDNA haplotype). Among the red deer and sika in analysis 2, 86/606 or 14.2% of sika‐like animals (*Q* < 0.5) sampled had red deer mtDNA and one (0.04%) of the red deer‐like animals (*Q* > 0.5) had sika mtDNA. The majority of the individuals with discordant mitochondrial haplotypes were found in individuals collected from **Kintyre** (93%), the rest were from sites in the **North Highlands** (7%). Interestingly, within nuclear hybrids from Kintyre, the predominant haplotype carried differed between SK and WLA; 15 of 24 nuclear hybrids had sika mtDNA haplotype in SK, while all of the 102 nuclear hybrids in WLA carried the red deer mtDNA haplotype.

Of the animals showing mitochondrial discordance, almost half were mitochondrial hybrids, that is, they were pure of one species at their nuclear markers but had the mitochondrial haplotype of the other species, and all of these were pure sika at their nuclear markers but had red deer mitochondrial DNA. This represents a level of introgression beyond the 2–3 generations of backcrossing confidently detectable by our nuclear marker panel (Boecklen & Howard, 1997). As with the nuclear hybrids, the mitochondrial hybrids were spatially clustered. Within **Kintyre**, 15 cases were at SK and 11 were in and around WLA. Six mitochondrial hybrids were also identified in the *North Highlands*; Kildermorie (*n* = 4), Benmore (*n* = 1), and an unknown site in this region (*n* = 1). Kildermorie and Benmore lie approximately 80 km apart. Despite 11.4% of the 35 animals sampled from Kildermorie showing mitochondrial introgression, there were no nuclear hybrids identified at this site. However, one nuclear and one mitochondrial hybrid were sampled from the 50 animals obtained from Benmore. This suggests that at these sites, at least one hybridization event occurred sometime in the past, the offspring of which have backcrossed into sika. The nuclear hybrids detected at Torrachilty (above), which is approximately 48 km from Kildermorie, and 87 km from Benmore, are further evidence of such events.

#### 3.2.4 Analysis 3: Population structure assessment of all red deer and wapiti (*n* = 2,230)

After removing all sika and red‐sika hybrid individuals, a dataset comprising 2,230 red deer and wapiti was analyzed in structure. The log likelihoods calculated in structure revealed that, although there is relatively greater variation around the different values of *K* in this analysis, the most likely number of genetic populations in this dataset is *K* = 7, with an average Ln Pr (*X*|*K*) of −109,223.16 (*SD* = 16.34) and rate of change of 31.09 (Figure S7). While *K* = 7 is the most likely number of genetic populations, the objective of this analyses was to assess introgression between red deer and wapiti individuals. Due to the strong differentiation of Harris and Lewis red deer, in this analysis wapiti were differentiated from red deer at *K* = 4 and while not the most likely *K*, this is most appropriate *K* for answering our question (Figure S8). This analysis identified six of the seven red‐wapiti hybrids previously identified in analysis 1, namely two each from **Kintyre**,** North Highlands** and **Central Highlands** (the remaining animal from the **Hebrides**, which only just qualified as hybrid analysis 1, was classified as a pure red deer in analysis 3). Three of the six potential red‐wapiti hybrids had 90% probability intervals that spanned zero for wapiti; the same individual as identified in analysis 1 from the **Central Highlands** and two individuals from **Kintyre** had probability intervals that did not span 0. Allele frequencies for the analysis 3 population clusters at *K* = 4 are shown in Table S5.

### 3.3 The initial direction of hybridization between red deer and sika

**Figure 4:**
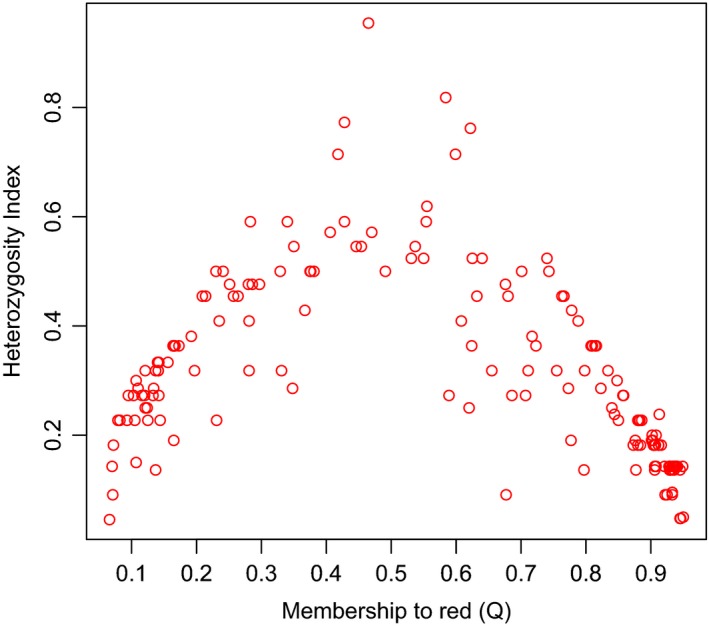
Plot of heterozygosity index (the proportion of loci at which an individual was heterozygous for red deer and sika alleles) of all hybrids against their membership to the red deer cluster (*Q*)

Based on the estimates of species‐specific allele frequencies generated from analysis 2 (Table S2), the majority of red‐sika hybrids had low heterozygosity between red and sika alleles. However, a single individual (WYM080) returned a heterozygosity index which may be consistent with an F1 (Figure 4). This potential F1 hybrid animal, shot at WLA, had a *Q* value of 0.465 and was heterozygous for a red deer and a sika allele at 21 of 22 markers with a single locus (RM95) homozygous for two sika alleles (the posterior estimate of the frequency of null alleles at this locus is 0.035 and 0.074 in red and sika respectively). This animal had a red deer mitochondrial haplotype, so if it was an F1, the father was a sika and the mother was a red deer.

## 4 DISCUSSION

Our data reveal very little influence of wapiti in Scottish or Lake District red deer, in that only three individuals from Kintyre and the Central Highlands had confirmed wapiti introgression. In contrast, we have documented a second area of extensive introgression between red and sika in Kintyre. We have also demonstrated that in scattered parts of the North Highlands, there are small numbers of individuals which look like red deer or sika but they have introgressed genes from the other species, while in the Lake District, very few phenotypically red deer carry traces of sika. The Hebrides populations of red deer appear free of sika introgression to date.

Assessment of the extent of introgression between all three *Cervus* species (analysis 1) and between red and wapiti (analysis 3) was complicated by the fact that a strongly divergent red deer population, sampled from the Hebridean island of Harris and Lewis, was identified by STRUCTURE (at *K* = 3) before differentiation of wapiti (at *K* = 4) from red deer. This is perhaps surprising given the mtDNA distinctness of wapiti (Kuwayama & Ozawa, 2000; Ludt et al., 2004) and their origin in Eastern Asia and North America. Two factors may have contributed to this scenario: The microsatellite loci were not actually selected to differentiate red deer and wapiti and only 10/22 do so strongly; second, the red deer on Harris and Lewis may have been separated from other red deer in our sample for a long time, with drift leading to divergent allele frequencies. The marked genetic differentiation of Harris and Lewis was also reported by Pérez‐Espona et al. (2013), with red deer from these locations also presenting the lowest allelic richness of all Scottish red deer populations analyzed, suggesting that the red deer population on this island might have originated from a small number of individuals. A recent mtDNA survey of red deer from Scottish archaeological sites up to 7,500 years BP found that samples from the Outer Hebrides and Orkney (where they are now extinct) had haplotypes not found in the rest of Scotland and suggested that human‐mediated transfer from a source beyond Scotland was likely (Stanton et al., 2016). Together these observations suggest further work on the modern red deer of Harris and Lewis would be useful, in particular, a survey of mtDNA variation. Nevertheless, in this study, we combined the two red deer clusters to represent a single red deer species unit. Taking this approach, evidence for low‐level wapiti introgression was found in seven red deer‐like animals when analyzed in the context of all samples (analysis 1). Once the data were re‐analyzed without sika and red‐sika hybrid animals (analysis 3), only six of these animals retained *Q* values that fell within our definition of a hybrid, one of which was from Mamore, which historically held a large wapiti herd (Whitehead, 1964). However, only three of the six had 90% probability intervals that did not overlap with *Q* = 1. These findings either represent genuine wapiti introgression from previous hybridization events or are an artifact of ancestral polymorphism and the limited resolution of our marker panel. This low impact of wapiti on Scottish red deer supports the conclusions of a previous study (Pérez‐Espona et al., 2013), with which this study shares 235 individuals but only two loci. Previous studies have also found no evidence for introgression of the either wapiti mtDNA or Y chromosome among red deer in the Central Highlands (Pérez‐Espona, Pérez‐Barbería, et al., 2009; Pérez‐Espona et al., 2011).

It is evident from analysis of all samples (analysis 1*, n* = 2,943), and red deer and sika only (analysis 2, *n* = 2,887), that there is introgression between red deer and sika in some parts of Scotland. In particular, hybrids are abundant in two regions of Kintyre (WLA and SK) where 50% and 58% of the individuals analyzed were nuclear or mitochondrial hybrids, respectively, and evidence of introgression was also found in adjacent sites. While the hybrid swarm at WLA has been documented previously (Senn & Pemberton, 2009), the extensive hybridization found at SK has only become apparent with the additional samples collected for this study. A major difference between these two regions is the absence of the sika mtDNA haplotype among hybrids in WLA and its prevalence in SK. A possible explanation for this may be that the relative densities of the deer species in the two areas were different when hybridization and introgression started. The introduction to Carradale, Kintyre, in 1893, was at a time when red deer were colonizing the peninsula from the north but were rare or absent in the south. Contact and hybridization probably occurred around 40 years ago (Senn & Pemberton, 2009; Senn, Barton et al., 2010), by which time the southern sika population had probably built up to the extent that roving males spread north into a red deer population of both sexes still expanding southwards (Ratcliffe, 1987; Senn & Pemberton, 2009). In consequence, any F1 hybrids produced were initially most likely to backcross into red deer, given their higher local population density compared to sika. The fact that there are mitochondrial hybrids which are pure sika at the nuclear markers but carry red deer mtDNA suggests that extensive backcrossing into sika also subsequently occurred. In contrast, in SK, sika were locally abundant but red deer probably rare (Ratcliffe, 1987; Senn & Pemberton, 2009; Whitehead, 1964), such that any hybrids generated here were more likely to backcross into sika. More recently, escapes known to have occurred from a red deer farm in SK would likewise have found themselves in sika‐dominated territory (K. McKillop pers. comm.).

Regarding other parts of Scotland, only 0.53% of the individuals collected from the North Highlands were identified as nuclear red‐sika hybrids and 1.05% as mitochondrial hybrids. This is consistent with extensive low‐level introgression among sika‐like animals previously reported throughout Ross‐shire and Sutherland based on fewer and less diagnostic loci than this study (Swanson, 2000). In the present study, the identification of sika‐like animals showing mitochondrial discordance from sites otherwise relatively free of nuclear introgression (e.g., Kildermorie) highlights that there has been quite widespread introgression via repeated backcrossing which is undetectable by the present nuclear marker set and which has failed to break down the general pattern of assortative mating between the two species.

There was no evidence for hybrids among phenotypically red deer sampled across the Central Highlands, even among 50 animals collected from Ralia, Abernethy, Inshriach, and Kinveachy on the western borders of the Cairngorm National Park into which sika are expanding. This is consistent with the lack of sika mtDNA sequences in Central Highland red deer (Pérez‐Espona, Pérez‐Barbería, et al., 2009). Similarly, no evidence for red‐sika hybrids was found in the Hebrides, confirming the integrity of the red deer refugium.

Within the Lake District, only three of 137 (2.2%) red deer‐like animals were found to be hybrids, and there was no evidence of sika mtDNA in the samples. While there is a documented history of phenotypically hybrid individuals in this region (Lowe & Gardiner, 1975; Whitehead, 1964), none were sampled in this study and it is possible that increased culling pressure on conspicuous hybrids by the Lake District Deer Control Society in the 1970s may have reduced introgression in the area.

A single putative modern F1 was identified at WLA which, if true, was the result of a sika male mating a red deer female. No F1 was identified at any other site, precluding us from making any general comments on the direction of hybridization events except that, as previously inferred (Goodman et al., 1999), they are very rare. What we can say is that introgression can subsequently proceed in either direction to create individuals with Q values across the spectrum (as seen at WLA and SK), but mitochondrial introgression is nearly always by red deer mtDNA into sika‐like animals (*Q* < 0.5), with only one instance (from SK) of sika mtDNA in a red‐like animal (*Q* > 0.5). A survey of 471 red deer and sika on the island of Ireland using identical methods (Smith, Carden, Coad, Birkitt, & Pemberton, 2014), gave similar results: In the two areas with detected hybrids (Counties Wicklow and Cork), no F1 hybrids were found. In the well‐known hybrid swarm in County Wicklow, the nuclear markers show introgression in both directions, but mtDNA introgression was only from red into sika, and, as in Scotland, several mitochondrial hybrids (i.e., pure sika at nuclear markers but with red deer mtDNA) were found. In County Cork, where hybridization is more recent, all hybrids detected were sika‐like but with red deer mtDNA. The persistent appearance of red deer mtDNA in otherwise pure sika (examples in WLA, SK, Kildermorie, Benmore, and Wicklow) but not the reciprocal (i.e., sika mtDNA in pure red deer) suggests an asymmetry in the initial hybridization event (i.e., more often sika male with red deer female) or in the subsequent backcrossing process (hybrid female with sika male), a scenario concordant with the findings of (McDevitt et al., 2009) in Ireland and Senn and Pemberton (2009) in Kintyre. However, we cannot rule out the possibility that the red deer mtDNA is also favored by selection (Toews & Brelsford, 2012). Cytonuclear disequilibria due to asymmetric hybridization and/or selection have been observed in many other hybrid systems such as reef fishes (Hexagrammidae) (Crow et al., 2007), water striders (*Limnoporus dissortis* and *L. notabilis*) (Abe, Spence, & Sperling, 2005), Northern and Southern lineages of field vole (*Microtus agrestis*) (Beysard, Perrin, Jaarola, Heckel, & Vogel, 2012) and forest and savanna elephant ((*Loxodonta cyclotis* and *L. africana*) (Roca, Georgiadis, & O'Brien, 2005).

Overall, while introgression from wapiti is not currently a threat to red deer in Scotland, largely due to the modest numbers introduced and their failure to thrive, hybridization with sika is a threat, although it appears to be rather unpredictable as to where it occurs and more importantly, what the consequences are. In some areas, hybridization is followed by a breakdown of assortative mating and the generation of a hybrid swarm (e.g., WLA and SK), in others it is followed by repeated backcrossing into one or both parental species (e.g., the North Highlands and the Lake District) and assortative mating among parental (or parental‐like) species continues. The system may be perhaps best described as a “mottled” hybrid zone, in which the occurrence of hybridization is determined by demographic and environmental stochastic forces (Hauffe & Searle, 1993; Senn & Pemberton, 2009). For introgression to occur, a male of one species has to encounter, defend, and mate with a receptive female of the other species (an event which all the evidence suggests has very low probability), and the hybrid offspring has to survive and itself reproduce, events which may also have modest probabilities, especially for males. A somewhat similar system was found between races of house mice (*Mus musculus domesticus*)*,* due to a complex combination of extinction, re‐colonization and selection in response to stochastic patterns of flooding (Hauffe & Searle, 1993).

A major reason to study red‐sika hybridization is to guide future management of the situation. Before making detailed management suggestions, there are a number of points with practical implications that are worth noting. First, although our data for Scotland are not encouraging, it is possible for red deer and sika to coexist without hybridization and introgression, as exemplified by the population centered on Killarney, Ireland (Smith et al., 2014). Explanations for why hybrids do not occur in Killarney include that there is an unusually large physical difference in body size between red and sika in the area, and that there are sufficient females of both species present. Second, and related, it is widely hypothesized that hybridization occurs when dispersing males of one species find themselves in an area without females of the other species. Although there is no direct evidence for this, as sika have spread through Scotland, dispersing male sika may arrive in an area several years before sika females (Goodman et al., 1999; Swanson, 2000) and there are a number of instances of phenotypic hybrids being observed during or soon after this phase (Livingstone, 2001). Third, identification of hybrids from phenotype is very difficult in the field, making efficient selective culling difficult. In both Kintyre and those parts of Ireland where hybrids occur (Counties Wicklow and Cork), stalkers misclassify appreciable numbers of individuals as red or sika when they are genetically hybrid (Senn & Pemberton, 2009; Smith et al., 2014). Fourth, nevertheless, culling by shooting is the most practical tool available by which the situation may be managed. Deer fencing is very expensive over the large distances and rugged landscapes involved in Scotland and has limited effectiveness over time and where snow accumulates, as in many parts of Scotland. Moreover, in the area where we might be most concerned to contain hybrids by fencing, Kintyre, deer are quite frequently seen swimming between land masses (Kevin McKillop. Pers. Comm.). Live capture and testing followed by selective culling based on genotypes, as is suggested for the endangered bontebok (*Damaliscus pygargus pygargus*) being introgressed by common blesbok (*D. p. phillipsi*) on South African game ranches (van Wyk et al., 2017) is impractical in the Scottish context, as is any contraceptive method where delivery requires live capture. Contraceptive methods delivered in food would be very difficult to deliver in a targeted manner, given the presence of red deer, non‐*Cervus* deer and other mammals. Although shooting is thus the most practical tool for deer management, it should also be noted there is probably an optimum level of culling that minimizes emigration—many deer managers believe that shooting at a population too hard can actually encourage dispersal, meaning that the spread of hybrids could be exacerbated.

Preventing further hybridization and introgression involves a number of steps (1) prevention of initial hybridization events (2) prevention of breeding by F1 and introgressed individuals and (3) prevention of the spread of heavily introgressed animals out of Kintyre and into the rest of mainland Scotland. In addition (4) the red deer refugium in the Hebrides should be maintained free of sika introgression. To prevent initial hybridization, the best evidence suggests that selective culling of pioneering sika males, which have dispersed out of areas with sika females and into areas with only red females, is the best strategy. To prevent breeding by F1s and introgressed individuals, extreme vigilance for and culling of individuals with intermediate characteristics is required. With regard to the hybrids in Kintyre, the swarm at WLA is the furthest north and in greatest danger of sending dispersing males into the rest of mainland Scotland. Although a previous study of Kintyre (conducted before the SK hybrid swarm became apparent) showed no evidence that either the proportion of recent hybrids or the levels of introgression had increased over a period of 15 years (Senn, Barton et al., 2010), effective emigration (i.e., leading to successful mating) could occur at any time. Deer managers in North Kintyre, the adjacent Cowal peninsula, and in Argyll in general should therefore be especially vigilant for sika and intermediate animals moving north and east from WLA. For all three of these suggested actions, improved training of deer managers on the phenotypic appearance of sika and hybrids compared with red deer would be highly desirable.

Although in much of Scotland where sika and red co‐occur we found little evidence of hybridization and introgression, it would be wrong to conclude that introgression is consequently rare. The fact that we sampled a small fraction of the population of each species, that 22 nuclear loci can only detect 2–3 generations of backcrossing with any reliability (Boecklen & Howard, 1997) and the existence of scattered individuals in the North Highlands which are apparently pure sika but with red mtDNA all suggest that there may be many more advanced backcrosses present that we did not detect. The only way to reveal whether this is the case would be to use a large panel of markers, probably single nucleotide polymorphisms, (SNPs), giving reliable estimates of an individual's genetic status after many more generations of backcrossing. Bearing in mind the number of (fixed) markers required to define an individual as introgressed versus pure doubles with each generation of backcrossing, and that introgression may have been happening for several generations, several hundred or thousands of markers will be required. Second, although this study covers much of the Scottish *Cervus* deer range, there are gaps, of which the most notable is Dumfries and Galloway, in south west Scotland, where the red deer could have been introgressed by sika from the population that has expanded from the Dawyck, Peebleshire, introduction (Ratcliffe, 1987).

## CONFLICT OF INTEREST

None declared.

## AUTHOR CONTRIBUTION

SLS and MTW collected the 2008–2011 samples from the North Highlands and Kintyre, SPE collected the Central Highland samples and JMP collected the Hebrides samples. HVS contributed genotype data from her PhD work in Kintyre and from her survey of Scottish National Park deer, EH genotyped the Hebrides samples while SLS genotyped all other samples. SLS conducted all data analyses and drafted the MS, which all co‐authors helped to edit.
