# Supplementary material for: Introgression of exotic Cervus (nippon and canadensis) into red deer (Cervus elaphus) populations in Scotland and the English Lake District
Source: Ecol Evol. 2018 Jan 22;8(4):2122–34. doi: 10.1002/ece3.3767 (PMC5817139; doi:10.1002/ece3.3767)

Table S1. Details of the molecular markers used in this study from Senn & Pemberton (2009).

Loading panel	PCR plex	Locus name	Annealing temperature (° C)	Primer concentration (µM)	Label	Size Range (bp)	Deer linkage group	Primers (5'-3')
A	1	AGLA293*	58	0.06	PET	128-147	3	GTCTGAAATTGGAGGCAATGAGGC<br>CCCAAGACAACTCAAGTCAAAGGACC
	1	RM12§		0.06	VIC	116-151	9	CTGAGCTCAGGGGTTTTTGCT<br>ACTGGGAACCAAGGACTGTCA
	1	INRA6¶		0.1	NED	128-138	20	AGGAATATCTGTATCAACCTCAGTC<br>CTGAGCTGGGGTGGGAGCTATAAATA
	1	TGLA126*		0.12	6-FAM	100-105	-	CTAATTTAGAATGAGAGAGGCTTCT<br>TTGGTCTCTATTCTCTGAATATTCC
	2	IDVGA55¥	59	0.12	NED	191-221	4	GTGACTGTATTTGTGAACACCTA<br>TCTAAAACGGAGGCAGAGATG
	2	BM6438†		0.5	6-FAM	249-275	31	TTGAGCACAGACACAGACTGG<br>ACTGAATGCCTCCTTTGTGC
B	3	FSHB‡	56	0.5	6-FAM	180-210	1	CAGTTTCTAAGGCTACATGGT<br>TGGGATATAGACTTAGTGGC
	3	BOVIRBP**		0.25	NED	140-159	-	TGTATGATCACCTTCTATGCTTC<br>GCTTTAGGTAATCATCAGATAGC
	3	INRA131§§		0.12	PET	92-105	11	GGTAAAATCCTGCAAAACACAG<br>TGACTGTATAGACTGAAGCAAC
	3	BM4006†		0.06	VIC	85-95	-	CAATGTGCATTATTTCCAAAGTG<br>AGAAATAACTCTTTCTCCTTGGAGG
	Solo	RM188§	61	0.35	VIC	115-182	18	GGGTTCACAAAGAGCTGGAC<br>GCACTATTGGGCTGGTGATT
C	4	MM12¶¶	60	0.12	NED	89-104	26	CAAGACAGGTGTTTCAATCT<br>ATCGACTCTGGGGATGATGT
	4	BM757†		0.25	6-FAM	160-202	28	TGGAAACAATGTAAACCTGGG<br>TTGAGCCACCAAGGAACC
	4	OarFCB193¥¥		0.5	PET	103-143	5	TTCATCTCAGACTGGGATTCAGAAAGGC<br>GCTTGGAAATAACCCTCCTGCATCCC
	Solo	TGLA40*	56	0.25	6-FAM	91-108	10	GCTTCTCTGCCAACTAATATTATCC<br>CACCAGGTAAGCCCCTTATATATGT
	Solo	RM095†	54	0.12	VIC	118-147	31	TCCATGGGGTCGCAAACAGTGG<br>ATCCCTCCATTTGTTGTGGAGTT
D	5	TGLA127*	53	0.08	NED	161-192	20	CAATTGTGTGGTAGTTTGGACATTC<br>ACACTATTGCAAAAGGACCTCCAATT
	5	UWCA47††		0.5	6-FAM	225-240	29	GGAAAGTCCTTAGATGGAGGATTGT<br>TTGAGAACTTGTCCCGAGAGAA
	5	INRA5¶		0.25	VIC	129-143	30	CAATCTGCATGAAGTATAAATAT<br>CTTCAGGCATACCCTACACC
E	6	IDVGA29¥	54	0.25	VIC	136-156	-	CCCACAAGGTTATCTATCTCCAG<br>CCAAGAAGGTCCAAAGCATCCAC
	6	TGLA337*		0.5	PET	126-147	13	TTTGTTAAGGATAGTAGGCTACT<br>GCTCTTCCCTTGGTTTCCTTG
	Solo	RME25‡‡	54	0.5	6-FAM	151-207	12	AGTGGGTAAAGGAGCCTGGT<br>TTATTGATCCCAGCCTGTGC
MtDNA marker	-	SikaL3***<br>H16498§§§	52	0.5	-	430(sika)<br>350(red)	-	TTAAACTATTCCCTGACGCTT<br>CCTGAAGTAGGAACCAGATG

Table S2. Allele frequencies from analysis 2 (n = 2,886) at K = 2 and species-specific allele assignment to either red deer or sika. An allele was assigned to a species (red = red deer, green = sika) if its frequency was five-fold larger than the other species or the other species had a frequency for that allele of zero. The null allele frequency for each locus is also given.

Locus	% Missing data	Allele Size	Estimated allele frequency in red	Estimated allele frequency in sika	Allele species-specific assignment
**AGLA293**	**1.50%**	128	0.083	0.004	R
		144	0.773	0.021	R
		147	0.054	0.969	S
		Null	0.091	0.005	NA
**BM4006**	**0.40%**	85	0.001	0.974	S
		87	0.096	0.000	R
		93	0.731	0.024	R
		95	0.124	0.000	R
		Null	0.047	0.002	NA
**BM6438**	**1.20%**	249	0.547	0.001	R
		251	0.202	0.002	R
		253	0.098	0.000	R
		257	0.004	0.000	R
		259	0.000	0.080	S
		261	0.072	0.000	R
		265	0.000	0.273	S
		275	0.001	0.551	S
		Null	0.077	0.092	NA
**BM757**	**0.10%**	160	0.068	0.010	R
		162	0.543	0.003	R
		164	0.007	0.000	R
		172	0.000	0.916	S
		174	0.003	0.053	S
		179	0.053	0.000	R
		183	0.075	0.000	R
		185	0.044	0.001	R
		187	0.036	0.000	R
		189	0.002	0.000	R
		196	0.000	0.000	NA
		197	0.000	0.000	NA
		198	0.056	0.003	R
		200	0.069	0.000	R
		202	0.010	0.000	R
		210	0.004	0.000	R
		Null	0.029	0.013	NA
**BOVIRP**	**0.40%**	140	0.001	0.959	S
		142	0.000	0.016	S
		147	0.062	0.000	R
		149	0.056	0.000	R
		151	0.184	0.000	R
		153	0.364	0.008	R
		155	0.055	0.002	R
		157	0.203	0.000	R
		159	0.021	0.000	R
		163	0.000	0.000	NA
		Null	0.054	0.015	NA
**FCB193**	**2.00%**	101	0.002	0.000	R
		103	0.039	0.008	NA
		105	0.000	0.000	NA
		107	0.090	0.000	R
		109	0.161	0.000	R
		111	0.018	0.000	R
		113	0.236	0.000	R
		115	0.008	0.000	R
		118	0.039	0.000	R
		120	0.100	0.000	R
		122	0.101	0.001	R
		124	0.049	0.000	R
		126	0.011	0.028	NA
		128	0.011	0.051	NA
		130	0.060	0.000	R
		132	0.006	0.901	S
		134	0.004	0.002	NA
		140	0.003	0.000	R
		141	0.000	0.000	NA
		143	0.007	0.000	R
		Null	0.054	0.008	NA
**FSHB**	**0.70%**	179	0.000	0.009	S
		180	0.004	0.744	S
		181	0.000	0.111	S
		182	0.000	0.029	S
		184	0.048	0.000	R
		185	0.189	0.000	R
		186	0.001	0.000	R
		187	0.003	0.000	R
		188	0.126	0.000	R
		189	0.126	0.002	R
		190	0.002	0.028	S
		191	0.088	0.000	R
		192	0.018	0.000	R

		193	0.000	0.000	NA
		194	0.026	0.002	R
		195	0.000	0.000	NA
		196	0.009	0.000	R
		197	0.005	0.000	R
		198	0.072	0.000	R
		199	0.021	0.000	R
		200	0.000	0.000	NA
		201	0.006	0.000	R
		202	0.026	0.000	R
		203	0.021	0.007	NA
		204	0.010	0.000	R
		205	0.079	0.003	R
		206	0.034	0.000	R
		207	0.038	0.000	R
		210	0.010	0.000	R
		211	0.002	0.000	R
		Null	0.032	0.064	NA
**IDVGA29**	**1.40%**	136	0.668	0.018	R
		143	0.319	0.044	R
		156	0.002	0.911	S
		Null	0.012	0.028	NA
**IDVGA55**	**1.90%**	191	0.037	0.000	R
		193	0.087	0.000	R
		195	0.227	0.001	R
		197	0.284	0.000	R
		199	0.209	0.005	R
		202	0.023	0.000	R
		204	0.042	0.000	R
		208	0.000	0.001	S
		210	0.001	0.868	S
		212	0.000	0.064	S
		214	0.000	0.051	S
		217	0.037	0.000	R
		219	0.015	0.000	R
		221	0.000	0.000	NA
		Null	0.038	0.010	NA
**INRA005**	**0.10%**	124	0.000	0.036	S
		126	0.994	0.045	R
		129	0.000	0.002	S
		136	0.000	0.002	S
		143	0.000	0.914	S
		Null	0.006	0.001	NA
**INRA006**	**0.10%**	128	0.000	0.001	S
		130	0.000	0.947	S
		132	0.040	0.000	R
		134	0.682	0.042	R
		136	0.245	0.004	R
		138	0.010	0.000	R
		Null	0.023	0.007	NA
**INRA131**	**0.00%**	87	0.000	0.000	NA
		92	0.042	0.000	R
		94	0.008	0.086	S
		98	0.590	0.002	R
		100	0.233	0.000	R
		102	0.071	0.000	R
		104	0.037	0.000	R
		106	0.000	0.779	S
		113	0.000	0.048	S
		115	0.000	0.011	S
		Null	0.018	0.073	NA
**MM012**	**0.10%**	89	0.742	0.104	R
		91	0.231	0.004	R
		93	0.000	0.835	S
		97	0.001	0.000	R
		104	0.000	0.000	NA
		Null	0.026	0.056	NA
**RM012**	**0.50%**	116	0.003	0.991	S
		120	0.005	0.000	R
		125	0.165	0.001	R
		127	0.051	0.000	R
		129	0.072	0.000	R
		131	0.083	0.000	R
		133	0.244	0.000	R
		137	0.016	0.000	R
		139	0.085	0.005	R
		141	0.085	0.000	R
		144	0.095	0.000	R
		151	0.039	0.000	R
		Null	0.057	0.002	NA
**RM188**	**0.80%**	115	0.021	0.000	R
		117	0.037	0.000	R
		121	0.000	0.000	NA
		123	0.042	0.000	R
		125	0.074	0.000	R
		127	0.411	0.003	R
		129	0.207	0.009	R
		131	0.033	0.000	R
		132	0.030	0.000	R
		133	0.001	0.000	R
		134	0.041	0.000	R
		137	0.041	0.000	R
		139	0.003	0.037	S
		141	0.000	0.009	S
		143	0.000	0.550	S
		161	0.000	0.205	S
		163	0.000	0.002	S
		176	0.000	0.027	S
		182	0.000	0.143	S
		Null	0.058	0.015	NA

**RM95**	**0.50%**	116	0.000	0.117	S
		118	0.054	0.000	R
		120	0.002	0.000	R
		122	0.010	0.796	S
		124	0.086	0.000	R
		126	0.041	0.000	R
		128	0.179	0.001	R
		130	0.302	0.011	R
		132	0.099	0.000	R
		134	0.006	0.000	R
		136	0.077	0.000	R
		138	0.088	0.000	R
		140	0.019	0.000	R
		142	0.002	0.000	R
		147	0.000	0.001	S
		Null	0.035	0.074	NA
**RME025**	**0.50%**	151	0.020	0.000	R
		155	0.064	0.000	R
		157	0.001	0.000	R
		159	0.003	0.000	R
		168	0.764	0.005	R
		170	0.106	0.007	R
		183	0.001	0.000	R
		193	0.000	0.976	S
		207	0.010	0.000	R
		Null	0.031	0.011	NA
**TGLA40**	**0.30%**	91	0.195	0.000	R
		96	0.003	0.000	R
		97	0.492	0.010	R
		98	0.000	0.000	NA
		99	0.042	0.000	R
		101	0.191	0.000	R
		102	0.002	0.000	R
		104	0.001	0.758	S
		106	0.000	0.155	S
		108	0.001	0.001	NA
		Null	0.073	0.076	NA
**TGLA126**	**0.00%**	100	0.001	0.350	S
		101	0.000	0.548	S
		105	0.935	0.057	R
		130	0.000	0.003	S
		132	0.002	0.000	R
		134	0.006	0.000	R
		136	0.003	0.000	R
		138	0.000	0.000	NA
		Null	0.052	0.042	NA
**TGLA127**	**0.20%**	161	0.000	0.603	S
		167	0.014	0.000	R
		169	0.321	0.006	R
		171	0.000	0.000	NA
		172	0.003	0.003	NA
		174	0.027	0.290	S
		176	0.018	0.000	R
		178	0.240	0.012	R
		180	0.053	0.000	R
		184	0.100	0.000	R
		186	0.072	0.000	R
		188	0.002	0.000	R
		190	0.065	0.000	R
		192	0.039	0.000	R
		Null	0.046	0.085	NA
**TGLA337**	**8.40%**	126	0.005	0.592	S
		128	0.000	0.039	S
		130	0.201	0.000	R
		132	0.112	0.000	R
		134	0.002	0.000	R
		136	0.255	0.006	R
		138	0.042	0.193	NA
		142	0.001	0.000	R
		145	0.234	0.001	R
		147	0.065	0.022	NA
		153	0.001	0.000	R
		155	0.003	0.016	S
		Null	0.079	0.131	NA
**UWCA47**	**0.50%**	225	0.030	0.000	R
		229	0.049	0.000	R
		231	0.867	0.082	R
		240	0.000	0.878	S
		Null	0.054	0.040	NA

Table S3. Genetic diversity indices for each of the 22 loci in our microsatellite marker panel in phenotypic red deer (n = 2167), sika (n = 527) and wapiti (n = 49) calculated in Cervus 3.0. Subscripts $_{r,\ s,\ w}$ represent parameters calculated in red, sika and wapiti datasets independently. Parameters are k, the number of alleles at each locus in each species, N, number of samples typed at each locus, H_o, observed heterozygosity, H_e, expected heterozygosity and Null, the frequency of null alleles at each locus, after Table 3 in Senn & Pemberton (2009).

**Locus**	**K_r**	**N_r**	**H_{Or}**	**H_{Er}**	**$Null_r$**	**K_s**	**N_s**	**H_{Os}**	**H_{Es}**	**$Null_s$**	**K_w**	**N_w**	**H_{Ow}**	**H_{Ew}**	**$Null_w$**
AGLA293	3	2125	0.194	0.282	0.189	3	526	0.095	0.119	0.121	3	49	0.163	0.190	0.061
BM4006	4	2156	0.335	0.405	0.106	4	527	0.097	0.124	0.130	1	49	0.000	0.000	NA
BM6438	7	2137	0.497	0.596	0.080	6	524	0.412	0.559	0.151	3	49	0.224	0.270	0.078
BM757	16	2166	0.612	0.668	0.048	11	525	0.147	0.187	0.150	7	49	0.857	0.819	-0.030
BOVIRP	10	2156	0.659	0.762	0.073	7	527	0.089	0.119	0.160	5	48	0.563	0.714	0.122
FCB193	20	2113	0.768	0.870	0.062	13	525	0.189	0.211	0.075	9	49	0.592	0.552	-0.058
FSHB	28	2149	0.831	0.900	0.040	15	525	0.274	0.378	0.195	5	49	0.633	0.552	-0.082
IDVGA29	3	2127	0.432	0.450	0.021	3	527	0.135	0.173	0.130	1	49	0.000	0.000	NA
IDVGA55	11	2112	0.731	0.801	0.045	9	526	0.245	0.275	0.054	2	49	0.469	0.504	0.031
INRA005	2	2163	0.010	0.014	0.119	5	527	0.173	0.188	0.039	1	49	0.000	0.000	NA
INRA006	6	2165	0.414	0.454	0.046	4	526	0.101	0.150	0.252	2	49	0.245	0.217	-0.060
INRA131	9	2167	0.535	0.574	0.037	8	527	0.235	0.298	0.121	3	49	0.510	0.532	0.010
MM012	5	2166	0.328	0.364	0.052	3	526	0.179	0.242	0.162	3	49	0.653	0.535	-0.123
RM012	12	2151	0.755	0.862	0.065	7	527	0.068	0.078	0.080	4	49	0.286	0.345	0.076
RM188	16	2147	0.633	0.748	0.087	14	524	0.594	0.646	0.039	4	49	0.224	0.316	0.177
RM95	14	2156	0.759	0.829	0.044	12	525	0.187	0.284	0.230	7	49	0.735	0.796	0.036
RME025	9	2158	0.326	0.358	0.052	3	524	0.086	0.113	0.152	3	49	0.347	0.408	0.082
TGLA40	10	2159	0.520	0.634	0.101	6	526	0.236	0.340	0.180	2	49	0.449	0.444	-0.010
TGLA126	7	2167	0.021	0.045	0.328	4	527	0.491	0.570	0.069	2	49	0.265	0.340	0.119
TGLA127	14	2163	0.707	0.800	0.062	8	526	0.338	0.486	0.183	4	49	0.408	0.497	0.088
TGLA337	12	1932	0.649	0.794	0.100	9	522	0.314	0.507	0.263	3	47	0.277	0.330	0.080
UWCA47	4	2153	0.141	0.178	0.111	3	527	0.114	0.171	0.202	1	49	0.000	0.000	NA

Table S4. Posterior allele frequencies from analysis of red and wapiti (n = 2943) at K = 4.

Locus	% Missing data	Allele Size	Estimated allele frequency in Wapiti	Estimated allele frequency in Sika	Estimated allele frequency in Red I	Estimated allele frequency in Red II
AGLA293	1.50%	128	0.002	0.004	0.105	0.000
		144	0.921	0.018	0.738	0.993
		147	0.033	0.974	0.064	0.001
		149	0.011	0.000	0.000	0.000
		Null	0.032	0.003	0.093	0.006
BM4006	0.40%	85	0.001	0.977	0.001	0.000
		87	0.057	0.000	0.070	0.358
		93	0.827	0.021	0.839	0.138
		95	0.077	0.000	0.086	0.502
		Null	0.038	0.001	0.004	0.002
BM6438	1.20%	249	0.403	0.001	0.583	0.426
		251	0.176	0.001	0.233	0.000
		253	0.028	0.000	0.120	0.001
		257	0.033	0.000	0.001	0.000
		259	0.000	0.080	0.000	0.000
		261	0.007	0.000	0.024	0.561
		263	0.102	0.000	0.000	0.000
		265	0.021	0.273	0.000	0.000
		275	0.008	0.551	0.000	0.000
		Null	0.222	0.094	0.038	0.011
BM757	0.10%	160	0.107	0.010	0.066	0.033
		162	0.354	0.001	0.510	0.958
		164	0.000	0.000	0.009	0.000
		172	0.000	0.919	0.000	0.000
		173	0.039	0.000	0.000	0.000
		174	0.001	0.053	0.003	0.000
		175	0.009	0.000	0.000	0.000
		177	0.034	0.000	0.000	0.000
		179	0.083	0.000	0.055	0.000
		183	0.021	0.000	0.092	0.000
		185	0.001	0.000	0.056	0.000
		187	0.199	0.000	0.017	0.003
		189	0.000	0.000	0.003	0.000
		192	0.011	0.000	0.000	0.000
		196	0.000	0.000	0.001	0.000
		197	0.000	0.000	0.000	0.000
		198	0.039	0.003	0.071	0.000
		200	0.039	0.000	0.087	0.000
		202	0.000	0.000	0.013	0.000
		210	0.000	0.000	0.005	0.000
		Null	0.061	0.013	0.012	0.005
BOVIRP	0.40%	140	0.008	0.961	0.000	0.000
		142	0.000	0.016	0.000	0.000
		145	0.052	0.000	0.000	0.000
		147	0.055	0.000	0.077	0.000
		149	0.265	0.000	0.030	0.016
		151	0.070	0.000	0.162	0.481
		153	0.263	0.007	0.421	0.027
		155	0.002	0.002	0.060	0.071
		157	0.098	0.000	0.192	0.401
		159	0.075	0.000	0.015	0.000
		161	0.028	0.000	0.000	0.000
		163	0.000	0.000	0.000	0.000
		Null	0.084	0.013	0.043	0.004
FCB193	1.90%	101	0.005	0.000	0.001	0.000
		103	0.002	0.008	0.049	0.000
		105	0.000	0.000	0.001	0.001
		107	0.023	0.000	0.111	0.000
		109	0.014	0.000	0.203	0.000
		111	0.139	0.000	0.002	0.004
		113	0.151	0.000	0.270	0.038
		115	0.000	0.000	0.010	0.000
		118	0.011	0.000	0.047	0.003
		120	0.250	0.000	0.054	0.303
		122	0.058	0.001	0.102	0.127
		124	0.001	0.000	0.061	0.000
		126	0.104	0.027	0.003	0.077
		128	0.041	0.051	0.003	0.045
		130	0.001	0.000	0.032	0.364
		132	0.012	0.903	0.001	0.034
		134	0.007	0.002	0.005	0.000
		140	0.029	0.000	0.000	0.000
		141	0.000	0.000	0.000	0.000
		143	0.008	0.000	0.009	0.000
		145	0.006	0.000	0.000	0.000
		150	0.023	0.000	0.000	0.000
		Null	0.115	0.009	0.035	0.003
FSHB	0.70%	179	0.000	0.009	0.000	0.000
		180	0.025	0.746	0.001	0.000
		181	0.000	0.111	0.000	0.000
		182	0.102	0.029	0.000	0.000
		184	0.023	0.000	0.061	0.000
		185	0.074	0.000	0.232	0.000
		186	0.000	0.000	0.001	0.000
		187	0.002	0.000	0.004	0.000
		188	0.135	0.000	0.104	0.294
		189	0.175	0.002	0.126	0.054
		190	0.015	0.028	0.001	0.002
		191	0.055	0.000	0.077	0.195
		192	0.000	0.000	0.022	0.000

		193	0.000	0.000	0.000	0.000
		194	0.000	0.002	0.033	0.000
		195	0.000	0.000	0.000	0.000
		196	0.001	0.000	0.011	0.000
		197	0.002	0.000	0.006	0.006
		198	0.041	0.000	0.071	0.106
		199	0.008	0.000	0.024	0.006
		200	0.004	0.000	0.000	0.000
		201	0.008	0.000	0.006	0.000
		202	0.000	0.000	0.033	0.000
		203	0.001	0.007	0.026	0.000
		204	0.068	0.000	0.000	0.026
		205	0.097	0.001	0.049	0.299
		206	0.005	0.000	0.041	0.000
		207	0.081	0.000	0.036	0.000
		210	0.000	0.000	0.012	0.000
		211	0.000	0.000	0.003	0.000
		Null	0.075	0.064	0.020	0.012
IDVGA29	1.40%	134	-0.031	0.112	0.000	0.000
		136	-0.535	0.524	0.016	0.697
		143	-0.300	0.150	0.043	0.298
		156	-0.043	0.025	0.914	0.000
		Null	-0.092	0.189	0.026	0.006
IDVGA55	1.90%	191	0.106	0.000	0.038	0.034
		193	0.012	0.000	0.109	0.000
		195	0.221	0.001	0.254	0.006
		197	0.252	0.000	0.298	0.294
		199	0.112	0.003	0.166	0.649
		202	0.000	0.000	0.029	0.000
		204	0.001	0.000	0.054	0.000
		208	0.001	0.000	0.000	0.000
		210	0.003	0.871	0.001	0.000
		212	0.000	0.064	0.000	0.000
		214	0.000	0.051	0.000	0.000
		217	0.246	0.000	0.008	0.008
		219	0.000	0.000	0.019	0.000
		221	0.000	0.000	0.000	0.000
		Null	0.044	0.008	0.023	0.007
INRA005	0.10%	124	0.000	0.036	0.000	0.000
		126	0.979	0.042	0.994	0.995
		129	0.000	0.002	0.000	0.000
		136	0.000	0.002	0.000	0.000
		143	0.000	0.917	0.000	0.000
		Null	0.020	0.001	0.005	0.005
INRA006	0.10%	128	0.003	0.000	0.000	0.000
		130	0.001	0.953	0.000	0.000
		132	0.001	0.000	0.051	0.000
		134	0.638	0.039	0.638	0.892
		136	0.180	0.003	0.288	0.098
		138	0.001	0.000	0.012	0.000
		Null	0.177	0.005	0.010	0.010
INRA131	0.00%	87	0.000	0.000	0.000	0.000
		92	0.111	0.000	0.051	0.000
		94	0.006	0.086	0.009	0.000
		98	0.446	0.001	0.567	0.890
		100	0.359	0.000	0.243	0.003
		102	0.006	0.000	0.090	0.000
		104	0.001	0.000	0.034	0.099
		106	0.000	0.780	0.000	0.000
		113	0.000	0.048	0.000	0.000
		115	0.000	0.011	0.000	0.000
		Null	0.069	0.073	0.008	0.008
MM012	0.10%	89	0.624	0.102	0.715	0.991
		91	0.262	0.003	0.270	0.004
		93	0.048	0.839	0.000	0.000
		97	0.000	0.000	0.001	0.000
		104	0.000	0.000	0.000	0.000
		Null	0.065	0.056	0.013	0.005
RM012	0.50%	116	0.001	0.992	0.004	0.000
		144	0.002	0.000	0.042	0.665
		129	0.047	0.000	0.084	0.001
		141	0.097	0.000	0.094	0.002
		133	0.125	0.000	0.293	0.000
		127	0.126	0.000	0.060	0.027
		139	0.049	0.005	0.081	0.180
		125	0.169	0.001	0.186	0.000
		137	0.008	0.000	0.020	0.000
		131	0.039	0.000	0.091	0.069
		151	0.208	0.000	0.018	0.012
		120	0.016	0.000	0.001	0.017
		Null	0.112	0.002	0.025	0.026
RM188	0.80%	115	0.005	0.000	0.025	0.002
		117	0.003	0.000	0.046	0.000
		121	0.000	0.000	0.001	0.000
		123	0.035	0.000	0.048	0.000
		125	0.092	0.000	0.078	0.018
		127	0.173	0.001	0.386	0.940
		129	0.051	0.009	0.259	0.001
		131	0.001	0.000	0.042	0.000
		132	0.270	0.000	0.001	0.009
		133	0.000	0.000	0.001	0.000
		134	0.109	0.000	0.051	0.000
		137	0.091	0.000	0.039	0.001
		139	0.001	0.037	0.004	0.000
		141	0.000	0.009	0.000	0.000
		143	0.001	0.551	0.000	0.000

		161	0.000	0.205	0.000	0.000
		163	0.000	0.002	0.000	0.000
		176	0.000	0.027	0.000	0.000
		182	0.000	0.143	0.000	0.000
		Null	0.166	0.015	0.020	0.029
RM95	0.40%	122	0.069	0.795	0.004	0.049
		132	0.281	0.000	0.082	0.011
		128	0.082	0.000	0.174	0.318
		136	0.103	0.000	0.044	0.355
		138	0.077	0.000	0.106	0.000
		130	0.239	0.010	0.350	0.004
		124	0.006	0.000	0.110	0.000
		147	0.000	0.001	0.000	0.000
		118	0.001	0.000	0.037	0.257
		140	0.032	0.000	0.019	0.000
		126	0.001	0.000	0.053	0.000
		134	0.000	0.000	0.007	0.000
		142	0.028	0.000	0.002	0.000
		116	0.000	0.117	0.000	0.000
		120	0.000	0.000	0.002	0.000
		144	0.013	0.000	0.000	0.000
		153	0.002	0.000	0.000	0.000
		Null	0.065	0.077	0.010	0.007
RME025	0.50%	132	0.090	0.000	0.000	0.000
		134	0.035	0.000	0.000	0.000
		136	0.006	0.000	0.000	0.000
		151	0.005	0.000	0.025	0.000
		155	0.001	0.000	0.081	0.000
		157	0.000	0.000	0.000	0.006
		159	0.000	0.000	0.004	0.000
		168	0.640	0.003	0.722	0.986
		170	0.024	0.009	0.130	0.000
		183	0.000	0.000	0.001	0.000
		193	0.001	0.980	0.000	0.000
		207	0.002	0.000	0.012	0.000
		Null	0.196	0.009	0.025	0.007
TGLA40	0.30%	91	0.160	0.000	0.225	0.000
		96	0.024	0.000	0.000	0.000
		97	0.155	0.008	0.531	0.722
		98	0.002	0.000	0.000	0.000
		99	0.060	0.000	0.052	0.001
		101	0.484	0.000	0.140	0.242
		102	0.000	0.000	0.003	0.000
		104	0.001	0.760	0.001	0.000
		106	0.000	0.155	0.000	0.000
		108	0.005	0.001	0.000	0.000
		Null	0.109	0.076	0.048	0.034
TGLA126	0.00%	100	0.001	0.351	0.001	0.000
		101	0.000	0.550	0.000	0.000
		104	0.032	0.000	0.000	0.000
		105	0.899	0.053	0.926	0.992
		130	0.000	0.003	0.000	0.000
		132	0.000	0.000	0.002	0.000
		134	0.000	0.000	0.008	0.000
		136	0.000	0.000	0.004	0.000
		138	0.000	0.000	0.001	0.000
		Null	0.066	0.041	0.058	0.007
TGLA127	0.20%	161	0.000	0.604	0.001	0.000
		167	0.001	0.000	0.018	0.000
		169	0.074	0.006	0.373	0.191
		171	0.001	0.000	0.000	0.000
		172	0.001	0.003	0.004	0.000
		174	0.041	0.290	0.027	0.007
		176	0.148	0.000	0.002	0.001
		178	0.188	0.010	0.205	0.710
		180	0.211	0.000	0.043	0.001
		182	0.004	0.000	0.000	0.000
		184	0.016	0.000	0.115	0.078
		186	0.063	0.000	0.082	0.000
		188	0.000	0.000	0.002	0.000
		190	0.162	0.000	0.056	0.006
		192	0.042	0.000	0.043	0.000
		Null	0.048	0.086	0.030	0.006
TGLA337	8.40%	111	0.037	0.000	0.000	0.000
		118	0.130	0.000	0.000	0.000
		126	0.026	0.594	0.002	0.001
		128	0.001	0.039	0.000	0.000
		130	0.301	0.000	0.153	0.471
		132	0.001	0.000	0.137	0.000
		134	0.000	0.000	0.002	0.000
		136	0.094	0.005	0.256	0.375
		138	0.004	0.193	0.051	0.000
		142	0.000	0.000	0.001	0.000
		145	0.191	0.000	0.247	0.124
		147	0.079	0.021	0.069	0.008
		153	0.001	0.000	0.001	0.000
		155	0.001	0.016	0.003	0.000
		Null	0.133	0.131	0.077	0.021
UWCA47	0.50%	225	0.229	0.000	0.006	0.000
		229	0.014	0.000	0.060	0.002
		231	0.726	0.079	0.895	0.967
		240	0.000	0.882	0.000	0.000
		Null	0.031	0.038	0.039	0.031

Table S5. Posterior allele frequencies from analysis of red and wapiti (n = 2230) at K = 4.

Locus	% Missing data	Allele Size	Estimated allele frequency in Red Cluster III	Estimated allele frequency in Red Cluster II	Estimated allele frequency in Red Cluster I	Estimated allele frequency in Wapiti
AGLA293	1.70%	128	0.000	0.028	0.123	0.001
		144	0.990	0.881	0.708	0.882
		147	0.001	0.017	0.080	0.039
		149	0.000	0.000	0.000	0.055
		Null	0.009	0.073	0.089	0.023
BM4006	0.50%	85	0.000	0.000	0.005	0.000
		87	0.358	0.092	0.065	0.001
		93	0.132	0.833	0.830	0.994
		95	0.509	0.067	0.096	0.001
		Null	0.001	0.007	0.003	0.003
BM6438	1.30%	249	0.423	0.657	0.540	0.006
		251	0.001	0.134	0.279	0.002
		253	0.002	0.056	0.134	0.001
		257	0.000	0.019	0.000	0.000
		261	0.565	0.047	0.015	0.032
		263	0.000	0.000	0.000	0.800
		265	0.000	0.001	0.000	0.121
		275	0.000	0.003	0.000	0.000
		Null	0.009	0.084	0.033	0.038
BM757	0.00%	160	0.030	0.132	0.049	0.001
		162	0.959	0.451	0.521	0.003
		164	0.000	0.000	0.012	0.000
		172	0.000	0.000	0.004	0.000
		173	0.000	0.000	0.000	0.231
		174	0.000	0.000	0.005	0.000
		175	0.000	0.000	0.000	0.048
		177	0.000	0.000	0.000	0.202
		179	0.000	0.065	0.055	0.001
		183	0.000	0.042	0.103	0.001
		185	0.000	0.023	0.064	0.001
		187	0.004	0.099	0.014	0.043
		189	0.000	0.001	0.003	0.000
		192	0.000	0.000	0.000	0.058
		196	0.000	0.000	0.001	0.000
		197	0.000	0.000	0.000	0.000
		198	0.000	0.062	0.062	0.202
		200	0.000	0.083	0.071	0.205
		202	0.000	0.001	0.017	0.000
		210	0.000	0.000	0.007	0.000
		Null	0.004	0.04	0.011	0.002
BOVIRP	0.60%	140	0.000	0.004	0.003	0.000
		142	0.000	0.000	0.000	0.000
		145	0.000	0.000	0.000	0.335
		147	0.000	0.018	0.083	0.340
		149	0.016	0.133	0.035	0.001
		151	0.481	0.150	0.156	0.049
		153	0.025	0.393	0.420	0.003
		155	0.069	0.087	0.042	0.001
		157	0.405	0.150	0.190	0.067
		159	0.000	0.035	0.018	0.001
		161	0.000	0.000	0.000	0.155
		163	0.000	0.000	0.000	0.000
		Null	0.003	0.028	0.053	0.048
FCB193	2.50%	101	0.000	0.002	0.002	0.000
		103	0.000	0.001	0.061	0.000
		105	0.000	0.000	0.001	0.000
		107	0.000	0.094	0.109	0.001
		109	0.000	0.058	0.233	0.001
		111	0.005	0.067	0.001	0.000
		113	0.038	0.214	0.287	0.001
		115	0.000	0.030	0.000	0.000
		118	0.004	0.013	0.056	0.001
		120	0.306	0.180	0.041	0.072
		122	0.127	0.114	0.095	0.021
		124	0.000	0.011	0.065	0.000
		126	0.081	0.001	0.003	0.657
		128	0.048	0.021	0.002	0.008
		130	0.352	0.080	0.011	0.001
		132	0.033	0.006	0.003	0.020
		134	0.000	0.013	0.001	0.022
		140	0.000	0.013	0.000	0.000
		141	0.000	0.000	0.000	0.000
		143	0.000	0.001	0.012	0.041
		145	0.000	0.000	0.000	0.028
		150	0.000	0.000	0.000	0.122
		Null	0.004	0.081	0.016	0.002
FSHB	0.80%	180	0.000	0.011	0.003	0.000
		182	0.000	0.000	0.000	0.621
		184	0.000	0.172	0.007	0.115
		185	0.000	0.115	0.255	0.108
		186	0.000	0.000	0.002	0.000
		187	0.000	0.011	0.000	0.009
		188	0.298	0.102	0.111	0.138
		189	0.053	0.147	0.137	0.001
		190	0.003	0.008	0.001	0.000
		191	0.197	0.065	0.079	0.001
		192	0.000	0.002	0.029	0.000

		193	0.000	0.000	0.000	0.000
		194	0.000	0.000	0.036	0.000
		195	0.000	0.000	0.000	0.000
		196	0.000	0.001	0.015	0.000
		197	0.005	0.004	0.007	0.000
		198	0.102	0.072	0.067	0.001
		199	0.005	0.005	0.031	0.000
		200	0.000	0.001	0.000	0.000
		201	0.000	0.006	0.007	0.000
		202	0.000	0.010	0.038	0.000
		203	0.000	0.011	0.026	0.000
		204	0.025	0.031	0.000	0.000
		205	0.301	0.050	0.060	0.001
		206	0.001	0.004	0.053	0.000
		207	0.000	0.099	0.021	0.000
		210	0.000	0.035	0.002	0.000
		211	0.000	0.000	0.003	0.000
		Null	0.007	0.038	0.011	0.001
IDVGA29	1.80%	134	0.000	0.000	0.000	0.972
		136	0.398	0.700	0.701	0.008
		143	0.600	0.284	0.289	0.004
		156	0.000	0.011	0.001	0.000
		Null	0.001	0.005	0.009	0.016
IDGVA55	2.50%	191	0.036	0.015	0.044	0.476
		193	0.000	0.024	0.123	0.001
		195	0.007	0.198	0.278	0.002
		197	0.300	0.203	0.332	0.513
		199	0.645	0.234	0.141	0.002
		202	0.000	0.001	0.038	0.000
		204	0.000	0.156	0.007	0.001
		210	0.000	0.003	0.003	0.000
		217	0.006	0.141	0.003	0.001
		219	0.000	0.000	0.025	0.000
		221	0.000	0.000	0.000	0.000
		Null	0.005	0.023	0.006	0.004
INRA005	0.20%	126	0.998	0.992	0.991	0.996
		143	0.000	0.000	0.002	0.000
		Null	0.002	0.007	0.007	0.004
INRA006	0.00%	128	0.000	0.001	0.000	0.000
		130	0.000	0.001	0.002	0.000
		132	0.000	0.031	0.054	0.001
		134	0.897	0.691	0.654	0.123
		136	0.095	0.248	0.262	0.873
		138	0.000	0.002	0.016	0.000
		Null	0.006	0.026	0.012	0.003
INRA131	0.00%	87	0.000	0.000	0.000	0.000
		92	0.000	0.013	0.060	0.611
		94	0.000	0.005	0.011	0.000
		98	0.891	0.637	0.541	0.280
		100	0.003	0.269	0.254	0.104
		102	0.000	0.037	0.098	0.001
		104	0.101	0.036	0.030	0.001
		106	0.000	0.000	0.002	0.000
		Null	0.003	0.003	0.005	0.003
MM012	0.00%	89	0.993	0.782	0.705	0.114
		91	0.004	0.209	0.275	0.625
		93	0.000	0.000	0.001	0.259
		97	0.000	0.000	0.002	0.000
		104	0.000	0.000	0.000	0.000
		Null	0.003	0.009	0.016	0.001
RM012	0.70%	116	0.000	0.000	0.009	0.000
		120	0.016	0.009	0.001	0.000
		125	0.000	0.112	0.212	0.001
		127	0.027	0.005	0.078	0.777
		129	0.001	0.079	0.086	0.001
		131	0.064	0.188	0.044	0.001
		133	0.001	0.206	0.314	0.009
		137	0.000	0.003	0.024	0.041
		139	0.186	0.024	0.097	0.154
		141	0.002	0.168	0.068	0.001
		144	0.669	0.062	0.032	0.001
		151	0.012	0.108	0.019	0.001
		Null	0.021	0.035	0.017	0.013
RM188	0.90%	115	0.002	0.007	0.029	0.000
		117	0.000	0.018	0.052	0.001
		121	0.000	0.000	0.001	0.000
		123	0.000	0.026	0.059	0.001
		125	0.016	0.151	0.054	0.001
		127	0.951	0.268	0.402	0.138
		129	0.001	0.254	0.229	0.002
		131	0.001	0.004	0.051	0.000
		132	0.007	0.118	0.000	0.020
		133	0.000	0.000	0.002	0.000
		134	0.000	0.001	0.063	0.789
		137	0.001	0.054	0.038	0.022
		139	0.000	0.005	0.003	0.000
		143	0.000	0.001	0.002	0.000

		161	0.000	0.000	0.000	0.000
		182	0.000	0.000	0.000	0.000
		Null	0.021	0.094	0.016	0.026
RM95	0.50%	118	0.260	0.034	0.035	0.001
		120	0.000	0.000	0.003	0.000
		122	0.050	0.008	0.006	0.300
		124	0.000	0.039	0.128	0.032
		126	0.000	0.021	0.053	0.001
		128	0.320	0.101	0.186	0.001
		130	0.004	0.433	0.310	0.002
		132	0.011	0.168	0.081	0.001
		134	0.000	0.000	0.009	0.000
		136	0.351	0.072	0.038	0.242
		138	0.001	0.061	0.121	0.183
		140	0.000	0.021	0.022	0.000
		142	0.000	0.000	0.003	0.149
		144	0.000	0.000	0.000	0.074
		153	0.000	0.000	0.000	0.009
		Null	0.004	0.041	0.005	0.005
RME025	0.40%	132	0.000	0.000	0.000	0.710
		134	0.000	0.000	0.000	0.227
		136	0.000	0.000	0.000	0.030
		151	0.000	0.004	0.033	0.001
		155	0.000	0.041	0.091	0.001
		157	0.006	0.000	0.000	0.000
		159	0.000	0.000	0.005	0.000
		168	0.988	0.898	0.690	0.007
		170	0.000	0.032	0.144	0.001
		183	0.000	0.001	0.002	0.000
		193	0.000	0.001	0.004	0.000
		207	0.000	0.006	0.014	0.000
		Null	0.005	0.017	0.018	0.022
TGLA40	0.40%	91	0.001	0.194	0.232	0.002
		96	0.000	0.011	0.000	0.000
		97	0.722	0.422	0.478	0.662
		98	0.000	0.001	0.000	0.000
		99	0.002	0.003	0.068	0.325
		101	0.247	0.239	0.169	0.002
		102	0.000	0.000	0.003	0.000
		104	0.000	0.001	0.003	0.000
		108	0.000	0.002	0.000	0.000
		Null	0.029	0.127	0.046	0.007
TGLA126	0.00%	100	0.000	0.000	0.002	0.000
		101	0.000	0.000	0.002	0.000
		104	0.000	0.000	0.000	0.191
		105	0.994	0.982	0.910	0.788
		132	0.000	0.000	0.003	0.000
		134	0.000	0.000	0.010	0.000
		136	0.000	0.000	0.005	0.000
		138	0.000	0.000	0.001	0.000
		Null	0.005	0.017	0.067	0.02
TGLA127	0.20%	161	0.000	0.000	0.002	0.000
		167	0.000	0.001	0.023	0.000
		169	0.187	0.264	0.369	0.002
		171	0.000	0.000	0.000	0.000
		172	0.000	0.000	0.005	0.000
		174	0.007	0.035	0.029	0.021
		176	0.000	0.067	0.002	0.000
		178	0.719	0.052	0.252	0.624
		180	0.001	0.082	0.051	0.319
		182	0.000	0.000	0.000	0.018
		184	0.075	0.141	0.086	0.001
		186	0.000	0.189	0.038	0.001
		188	0.000	0.000	0.003	0.000
		190	0.007	0.103	0.063	0.001
		192	0.000	0.020	0.054	0.001
		Null	0.003	0.044	0.022	0.011
TGLA337	0.20%	111	0.000	0.000	0.000	0.182
		118	0.000	0.000	0.000	0.758
		126	0.002	0.012	0.004	0.001
		128	0.000	0.000	0.000	0.000
		130	0.470	0.270	0.143	0.010
		132	0.000	0.020	0.163	0.001
		134	0.000	0.000	0.003	0.000
		136	0.372	0.219	0.236	0.002
		138	0.000	0.009	0.064	0.001
		142	0.000	0.000	0.001	0.000
		145	0.117	0.354	0.219	0.002
		147	0.008	0.054	0.081	0.001
		153	0.000	0.000	0.001	0.000
		155	0.000	0.001	0.004	0.000
		Null	0.03	0.06	0.08	0.041
UWCA47	0.60%	225	0.000	0.107	0.005	0.000
		229	0.002	0.020	0.073	0.001
		231	0.969	0.830	0.882	0.988
		240	0.000	0.000	0.000	0.000
		Null	0.03	0.043	0.04	0.011

Figures

Figure S1. Assessment of the most likely number of populations using Structure 2.3.3 analysis 1 of dataset containing all red deer, sika and wapiti (n=2,943) at K = 1 – 8. Two likelihood parameters are assessed; of which the results for a) the log-likelihood (with standard error) of the each value of K (number of populations) given the dataset and b) the rate of change in log likelihood between values of K. Both provide evidence that K = 2 is the most likely. The variation in the log likelihood generated during replicated simulations at the same value of K may be attributed to slight variation in the sampling (or "mixing") of the Markov chain, as part of the Bayesian analysis, when converging on the posterior distribution of each of the required parameters (Pritchard et al. 2000).

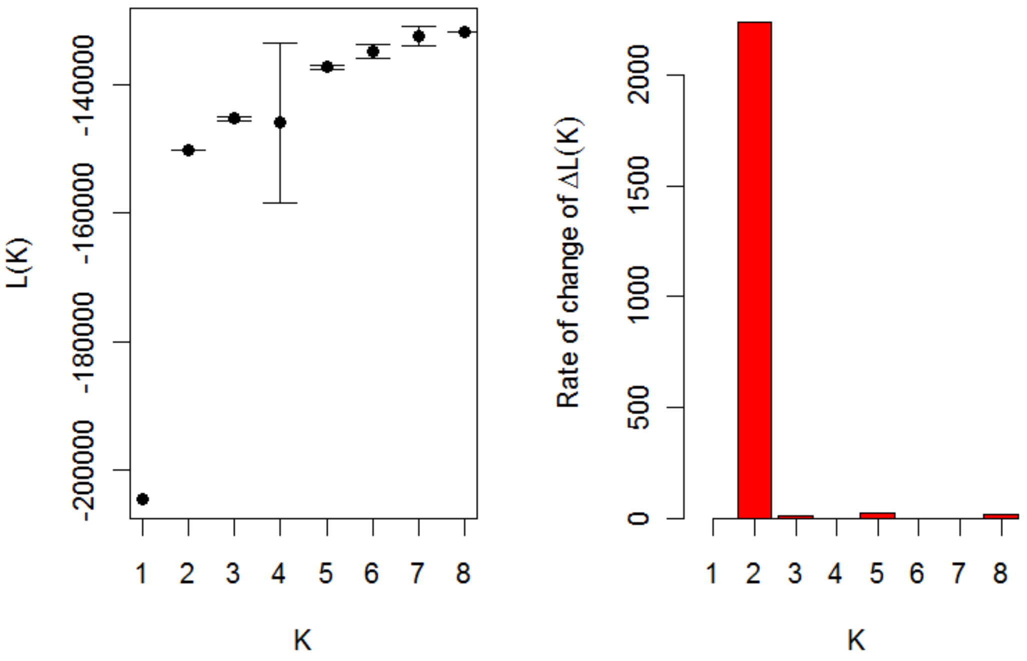

Figure S2. Bar chart showing the results of analysis 1 at K = 2 (n = 2943). It shows the proportion of an individual's nuclear genome attributable to red ancestry (shown in red) and the proportion attributable to sika ancestry (green), determined by the Q value on the y-axis against the county from which it was obtained on the x-axis.

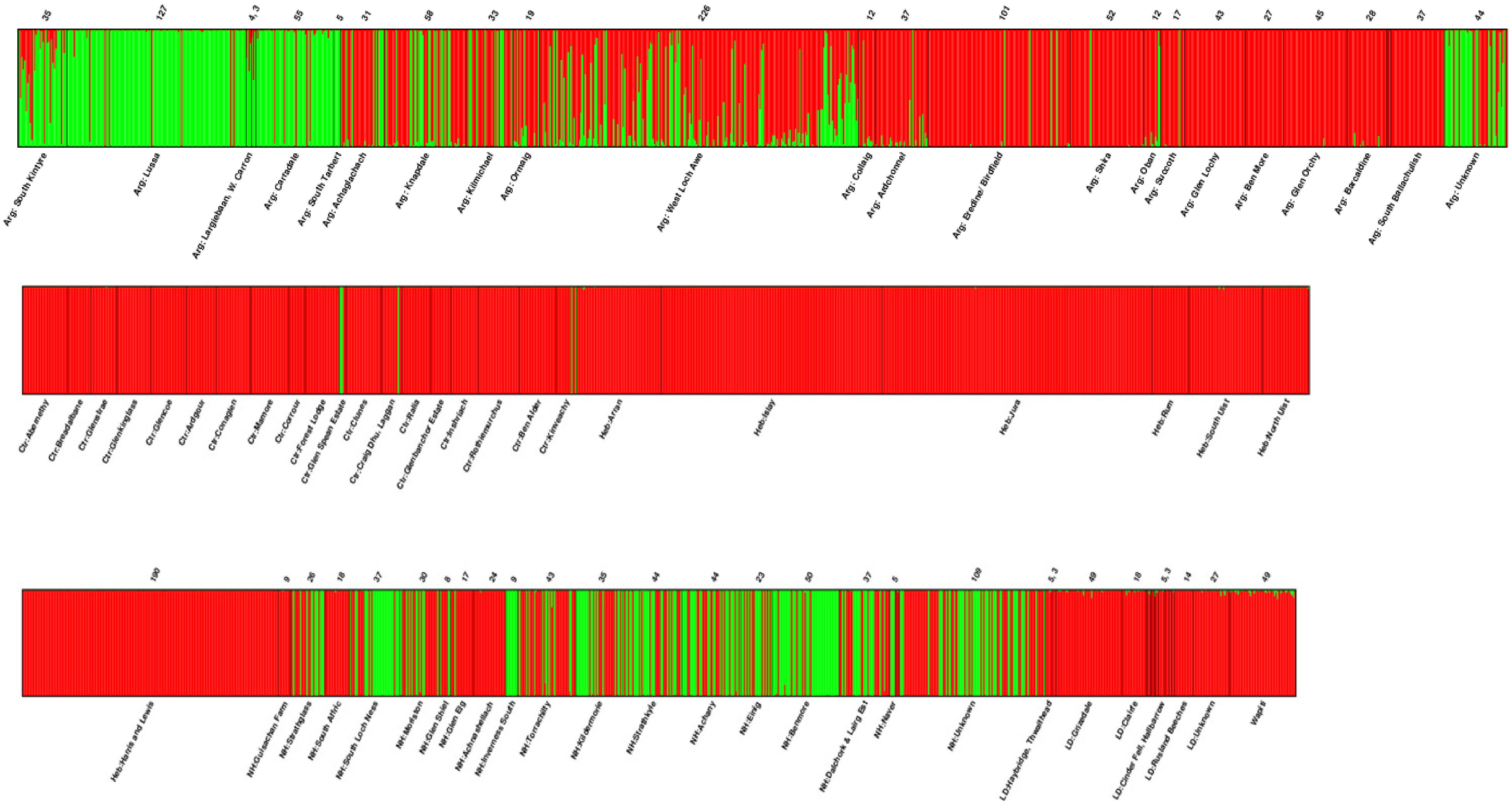

Figure S3. Bar chart showing the results of Structure from analysis 1 at K = 4 for each individual in the dataset (n = 2943). It shows the proportion of an individual's nuclear genome attributable to red ancestry (shown in red and pink) and the proportion attributable to sika ancestry (green) and to wapiti (blue), determined by the Q value on the y-axis against the population from which it was obtained on the x-axis. Scottish sites are plotted in an approximately south to north order, followed by the sample sites in the Lake District, Cumbria and lastly the wapiti controls. Abbreviations represent; Arg= Argyll, Ctr= Central highlands, Heb= Hebrides, NH= North Highlands and LD= Lake District, Cumbria.

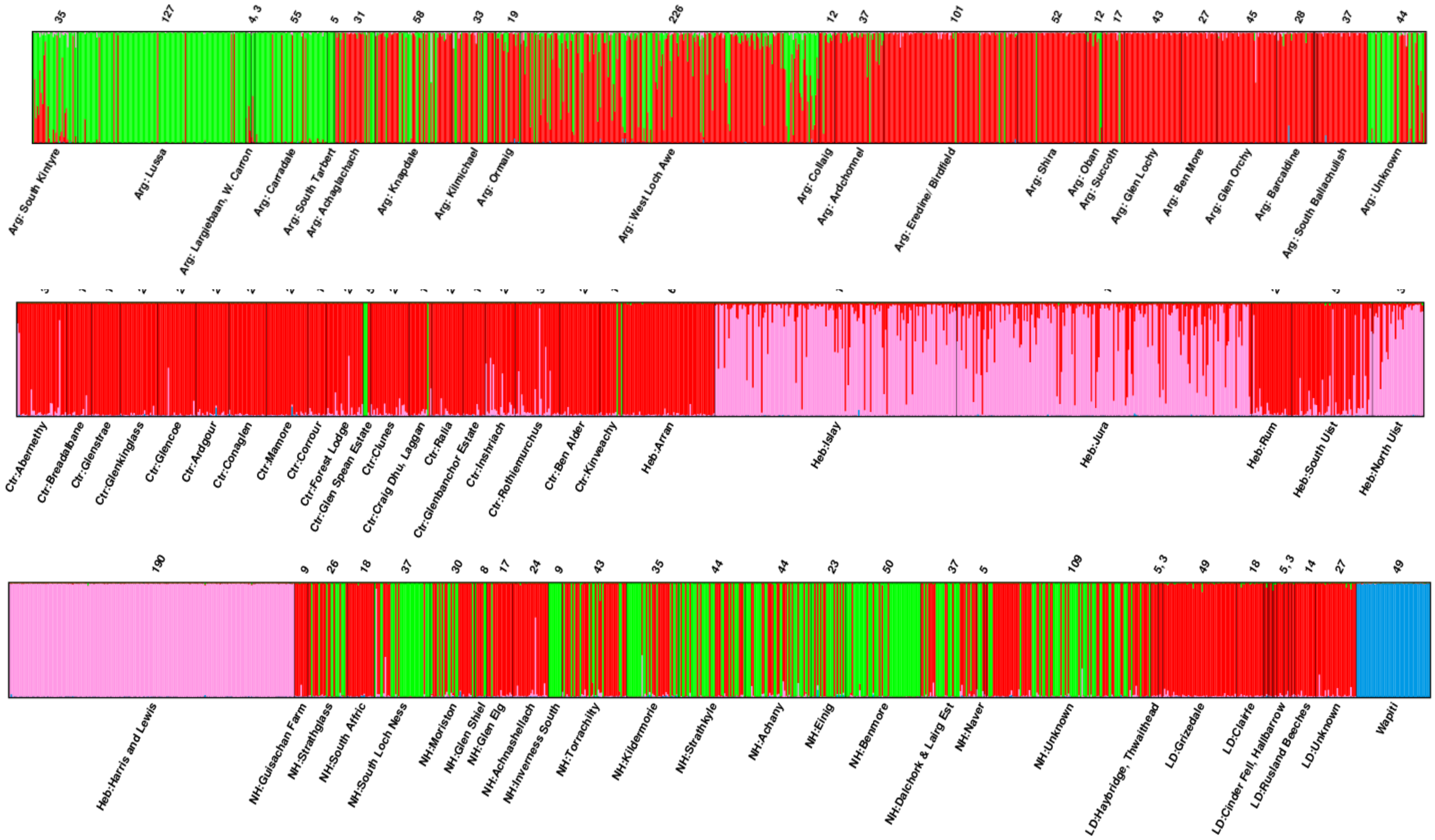

Figure S4. The proportion of inferred a) red, b) sika and c) wapiti ancestry determined by Q value generated in analysis 1 in Structure at K=4 (however red cluster I and II were combined for this figure) for the four regions in Scotland, in Cumbria and the wapiti control samples.

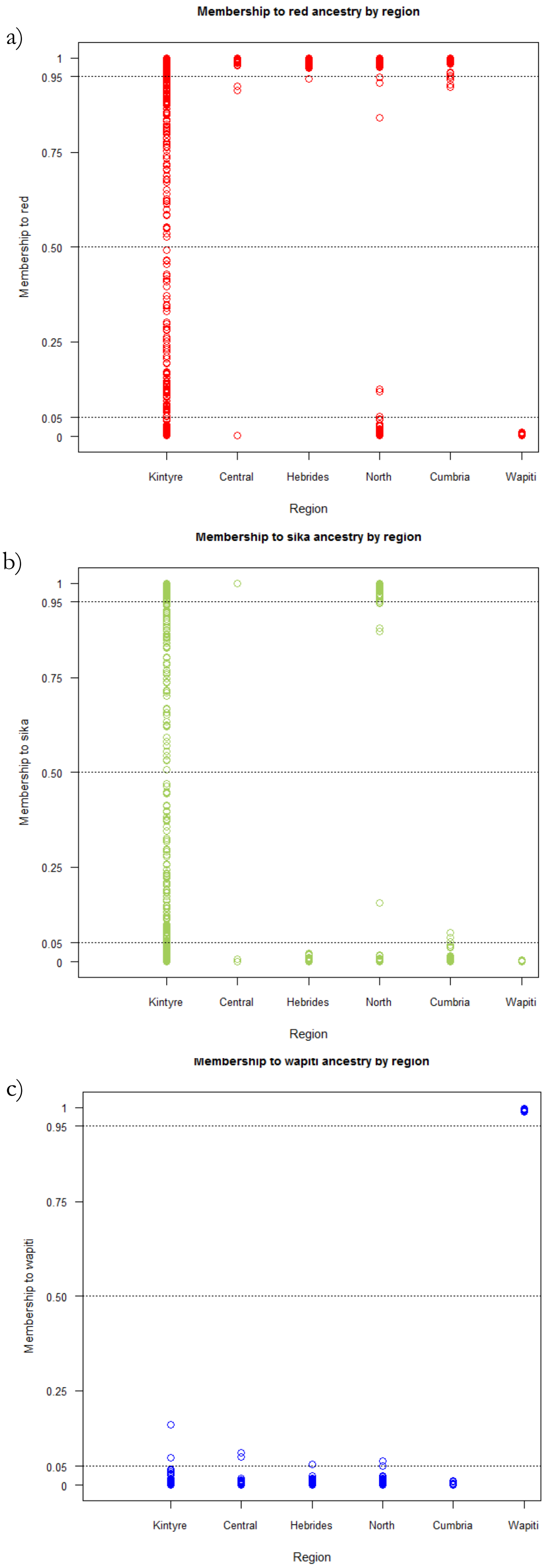

Figure S5. Assessment of the most likely number of populations using Structure 2.3.3 analysis 2 of dataset containing red deer and sika individuals only (n = 2,886) at K = 1 – 8. Two likelihood parameters are assessed; of which the results for a) the log-likelihood (with standard error) of the each value of K (number of populations) given the dataset and b) the rate of change in log likelihood between values of K. Both provide evidence that K = 2 is the most likely.

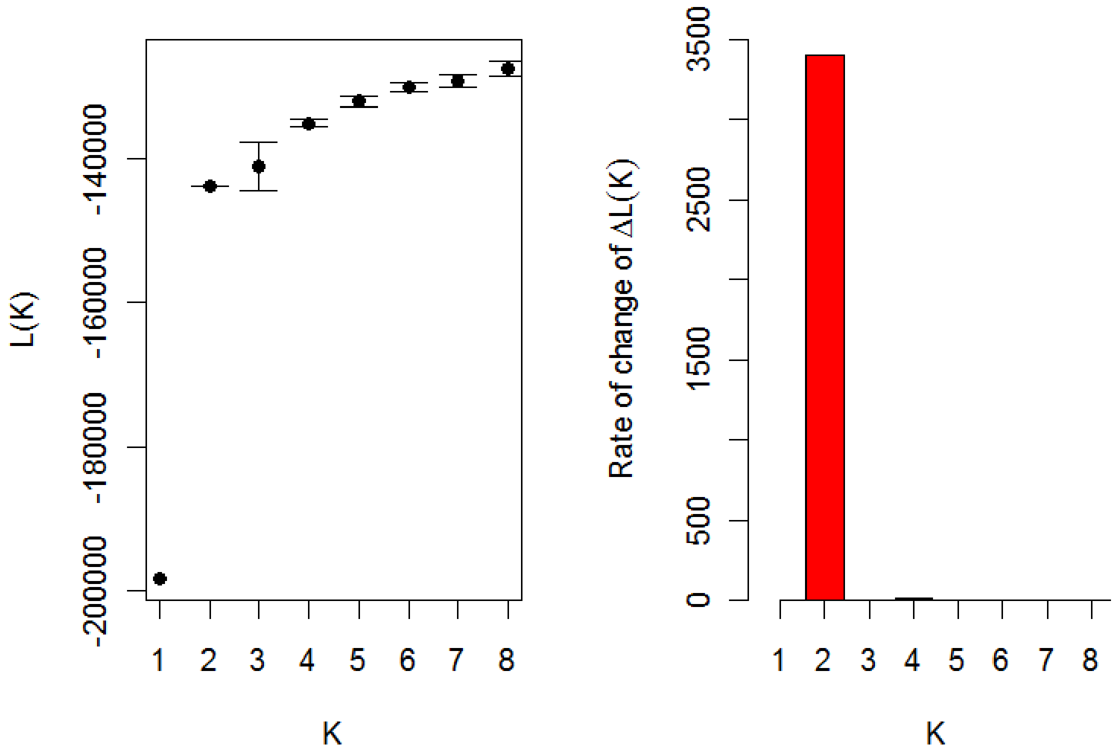

Figure S6. Bar chart showing the results of analysis 2 in STRUCTURE at K = 2 for each individual in the dataset consisting of red and sika animals only (n = 2886). The Q value on the y-axis indicates the proportion of an individual's nuclear genome attributable to red ancestry (shown in red) and the proportion attributable to sika ancestry (shown in green). A hybrid is defined as an animal with membership ancestry of $0.05 \leq Q \leq 0.95$ to both red and sika. Populations from where samples were collected are indicated in the x-axis (lower) and the number of animals sampled from each site on the upper x-axis. Scottish sites are plotted in an approximately south to north order, followed by the sample sites in the Lake District, Cumbria. Abbreviations represent; Kin= Kintyre, Ctr= Central highlands, Heb= Hebrides, NH= North Highlands and LD= Lake District, Cumbria.

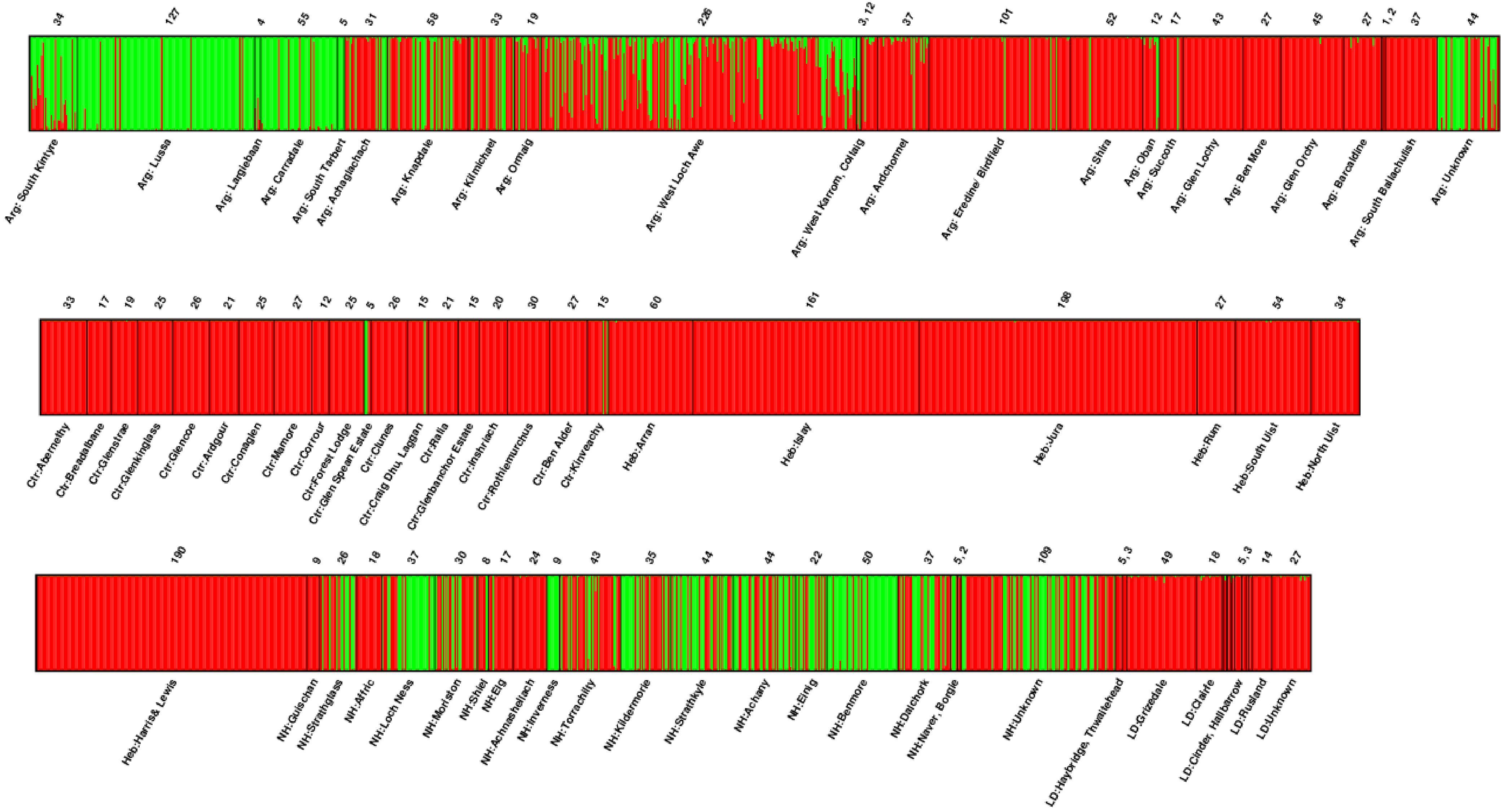
34
127
4
55
5
31
58
33
19
226
3, 12
37
101
52
12
17
43
27
45
27
1, 2
37
44
Arg: South Kintyre
Arg: Lussa
Arg: Lagiebaan
Arg: Carradale
Arg: South Tarbert
Arg: Achaglachach
Arg: Knapdale
Arg: Kilmichael
Arg: Ormaig
Arg: West Loch Awe
Arg: West Karrom, Coltaig
Arg: Ardchonnel
Arg: Eredine/ Birdfield
Arg: Shira
Arg: Oban
Arg: Succoth
Arg: Glen Lochy
Arg: Ben More
Arg: Glen Orchy
Arg: Barcaldine
Arg: South Ballachulish
Arg: Unknown
33
17
19
25
26
21
25
27
12
25
5
26
15
21
15
20
30
27
15
60
161
198
27
54
34
Ctr:Abernethy
Ctr:Breadalbane
Ctr:Glenstrae
Ctr:Glenkinglass
Ctr:Glencoe
Ctr:Ardgour
Ctr:Conaglen
Ctr:Mamore
Ctr:Corrour
Ctr:Forest Lodge
Ctr:Glen Spean Estate
Ctr:Clunes
Ctr:Craig Dhu, Laggan
Ctr:Ralia
Ctr:Glenbanchor Estate
Ctr:Inshriach
Ctr:Rothiemurchus
Ctr:Ben Alder
Ctr:Kinveachy
Heb:Arran
Heb:Islay
Heb:Jura
Heb:Rum
Heb:South Uist
Heb:North Uist
190
9
26
18
37
30
8
17
24
9
43
35
44
44
22
50
37
5, 2
109
5, 3
49
18
5, 3
14
27
Heb:Harris& Lewis
NH:Guischan
NH:Strathglass
NH:Affric
NH:Loch Ness
NH:Moriston
NH:Shiel
NH:Eig
NH:Achnashellach
NH:Inverness
NH:Torrachilty
NH:Kildermorie
NH:Strathkyle
NH:Achany
NH:Einig
NH:Benmore
NH:Dalchork
NH:Naver, Borgie
NH:Unknown
LD:Haybridge, Thwaitehead
LD:Grizedale
LD:Claife
LD:Cinder, Hallbarrow
LD:Rusland
LD:Unknown

Figure S7. Summary of simulation of dataset containing red and wapiti individuals only (n = 2230) across population K = 1 – 8. Two likelihood parameters are assessed; both the average likelihood itself at each value of K and the rate of change in the likelihood between values of K. Whilst K = 7 appears most likely, K = 4 is used to meet the objective of this analysis.

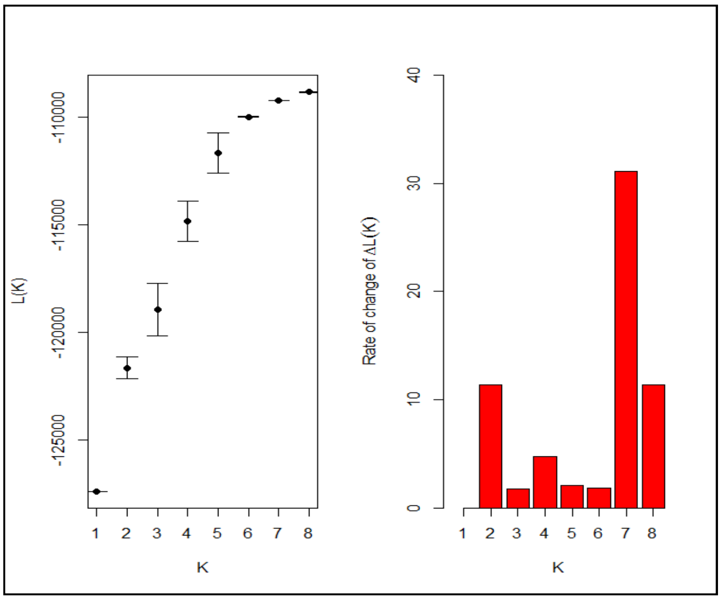

Figure S8. Structure results of red and wapiti in analysis 3 (n = 2230) at K = 4. It shows the proportion of an individual's nuclear genome attributable to three red clusters (pink, cream and brown) and the proportion attributable to wapiti ancestry (purple), determined by the Q value on the y-axis against the site from which it was obtained on the x-axis.

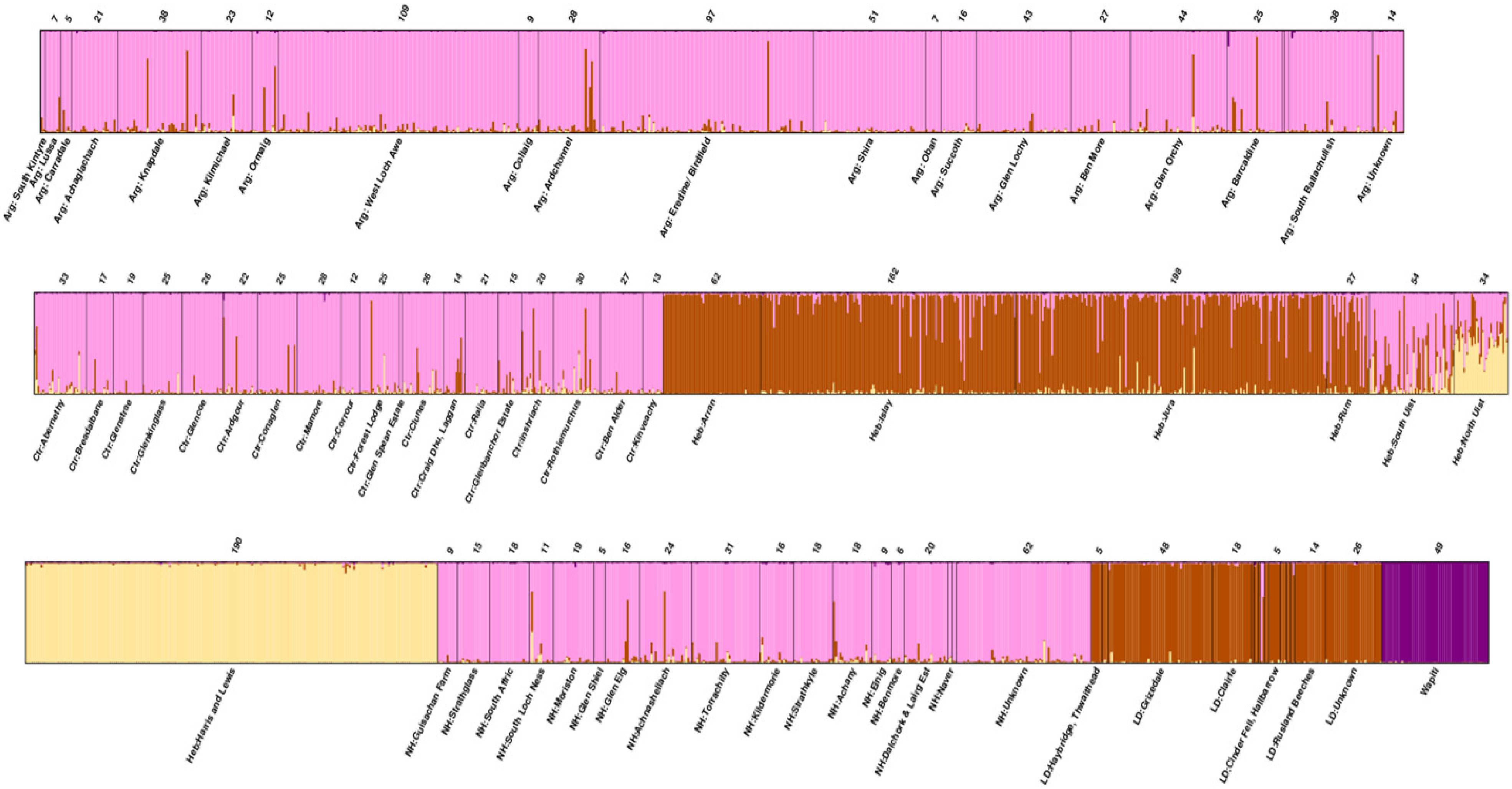

Supplement: Supplementary file 1 [file ECE3-8-2122-s001.pdf]
